# Revealing G4 Structures as Drug Targets: Exploring the Potential of Novel Therapeutic Agents

**DOI:** 10.34133/research.1363

**Published:** 2026-07-21

**Authors:** Ying Che, Yi Peng, Xiaolong Pan, Xiaoyun Hu, Yating Zhao, Yingqi Zhao, Caizhi Tian, Ming Yuan, Yuhang Yin, Rongquan Gan, Yang Li, Zhanlei Pei, Shiyin Liu, Zijian Li, Haobo Dong, Yue Tan, Guogui Sun, Huizhe Wu, Minjie Wei

**Affiliations:** ^1^ Department of Pharmacology, School of Pharmacy, China Medical University, Shenyang 110122, Liaoning, P. R. China.; ^2^ Liaoning Key Laboratory of Molecular Targeted Anti-Tumor Drug Development and Evaluation, Liaoning Cancer Immune Peptide Drug Engineering Technology Research Center, Key Laboratory of Precision Diagnosis and Treatment of Gastrointestinal Tumors, Ministry of Education, China Medical University, Shenyang 110122, Liaoning, P. R. China.; ^3^ Scientific Experimental Center, School of Pharmacy, China Medical University, Shenyang 110122, Liaoning, P. R. China.; ^4^ Department of Breast Surgery, North China University of Science and Technology Affiliated Hospital, Tangshan 063000, Hebei, P. R. China.; ^5^ Department of Chemoradiation, North China University of Science and Technology Affiliated Hospital, Tangshan 063000, Hebei, P. R. China.; ^6^ Liaoning Medical Diagnosis and Treatment Center, Shenyang 110000, Liaoning, P. R. China.

## Abstract

G-quadruplex (G4) structures represent noncanonical secondary formations found in DNA and RNA. Recent research reveals that G4s are abnormally enriched in various cancers, with closely linked to malignancy, metastatic potential, and prognosis. These structures have emerged as promising biomarkers for cancer with notable clinical relevance. Concurrently, investigations into compounds that selectively recognize DNA or RNA G4s as potential anticancer agents offer new avenues for early cancer diagnosis and therapeutic strategies. This review systematically analyzes the chemical structural characteristics, structure–activity relationships, anticancer effects, and mechanisms of G4-targeting molecules in cancer treatment. Additionally, it summarizes novel nucleic acid-based drugs targeting G4 structures, providing valuable insights for the future development of more specific and effective G4-targeted therapeutic agents.

## Introduction

In recent years, the incidence of cancer has steadily increased, presenting a substantial public health challenge that poses a serious threat to human health. According to data from the World Health Organization, cancer has emerged as one of the leading global causes of mortality [1]. Consequently, enhancing research efforts to elucidate the mechanisms underlying cancer development and advance effective treatment strategies is imperative. Nucleic acids, including DNA and RNA, are essential carriers of genetic information in living organisms [2]. Recent investigations have increasingly affirmed the potential of nucleic acids as viable targets for anticancer drug development [3,4]. Traditional nucleic acid-targeted anticancer drugs, such as nitrogen mustards and platinum-based compounds, are linked to the development of multidrug resistance and marked side effects [5,6]. Thus, there is an urgent need to identify and develop novel anticancer targets. Nonclassical secondary structures of nucleic acids, such as G-quadruplexes (G4s), hairpins, cruciforms, triplexes, and i-motifs, influence key biological processes like transcription and translation [7]. Among these, G4s are the most extensively studied, potentially regulating gene expression and maintaining genomic stability in cancer contexts [8]. Both DNA and RNA can fold into G4 structures in vivo [9,10]. These structures are widely distributed throughout the human genome and transcriptome [11]. Prior studies have documented the involvement of DNA G4 structures in regulating DNA replication, transcription, and genomic integrity maintenance [12]. Additionally, small molecule agents targeting these structures have garnered considerable research attention [13]. Recently, RNA G4s (rG4s) have attracted increased focus due to the abundance of guanine-rich (G-rich) sequences in RNA capable of forming numerous G4 structures. These structures are implicated in the regulation of both coding and noncoding RNA biosynthesis and have gained substantial interest for their potential roles in various pathophysiological processes in organisms [14,15]. This review discusses the structural and functional similarities and differences between DNA and RNA G4s and systematically summarizes recent advances in developing G4s as therapeutic targets for cancer.

## Structural Characteristics of DNA and RNA G4s

**Fig. 1.**
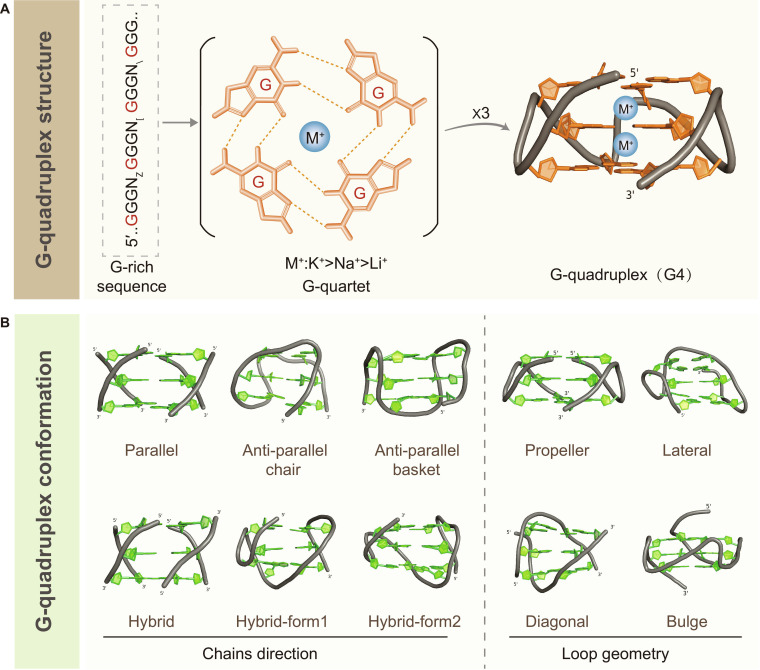
Structure and conformational diversity of G4s. (A) Guanine-rich sequences (motif…GGGN_x_GGGN_y_GGGN_z_GGG…) are stabilized by metal cations M (affinity: K^+^ > Na^+^ > Li^+^), forming planar G-quartets via Hoogsteen hydrogen bonding. Stacking of 2 or more G-quartets through π–π interactions yields G4 structures. In this context, N represents any nucleotide in loops connecting G-tracts, while x, y, and z indicate variable loop lengths. (B) G4 topology is classified by strand orientation (parallel, antiparallel chair, antiparallel basket, hybrid, hybrid-form1, hybrid-form2) and loop conformation (propeller, diagonal, lateral, bulge).

The G4 represents a highly stable secondary structure of nucleic acids, commonly observed in G-rich sequences [16]. Formation of a G4 structure typically necessitates the presence of 4 or more polyG (G-rich) regions. The canonical motif features 4 conserved guanine tracts separated by loop regions, arranged in the sequence GGGNxGGGNyGGGNzGGG, where N denotes any nucleotide, and x, y, and z indicate loop lengths ranging from 1 to 7 nucleotides. Variations in the number of guanine constituents can lead to the emergence of noncanonical G4 forms distinct from the classical motif [17]. Structurally, multiple guanine monomers link via Hoogsteen hydrogen bonds to create a planar, ring-like arrangement known as a G-quartet structure [18]. At physiologically relevant temperature (37 °C) and optimal K^+^ ion concentrations (100 mM), 2 guanine bases form G:G base pairs, resulting in a 2-G-quartet G4 [19]. Subsequent stacking of 2 or more G-tetrads through π–π interactions yields a higher-order structure comprising 4 polynucleotide strands, referred to as the G4 [14,20]. Notably, a strongly electronegative cavity forms between the 2 G-tetrad layers, and the structural stability of G4s is heavily influenced by the presence of specific cations (e.g., K^+^, Na^+^, and NH4^+^) within this cavity [21]. These cations facilitate electrostatic neutralization, structural stabilization, and coordination (Fig. 1A).

G4s exhibit marked structural diversity, influenced by various factors, including the type of central cation, the conformation of the glycosidic bond, the number and orientation of participating strands, the characteristics of the connecting loops, the total number of G4 units, and the nucleotide sequence composition [22]. This structural variability is reflected in their topological configurations, which can be classified into 3 main types—parallel, antiparallel, and hybrid conformations—based on the orientation of the DNA strands. The antiparallel conformation can be further divided into 2 subtypes: chair-like and basket-like structures [17]. Additionally, G4 structures can be categorized according to the glycosidic bond conformation of guanine residues (syn and anti) [22] and loop characteristics (propeller, diagonal, edge, and bulged loops) [23]. In summary, G4s demonstrate a high degree of conformational polymorphism, necessitating exploration of how these features manifest differently in DNA versus RNA. The following section examines the distinct folded conformations of DNA and RNA G4s and critically discusses emerging evidence regarding their direct and indirect cross-interactions (Fig. 1B).

## Folded Structures and Cross-Interactions between DNA and RNA G4s

### Folded structures of DNA and RNA G4s

The G4 structure in DNA can manifest either within a single strand of double-stranded DNA or between 2 separate G-rich strands, resulting in the formation of an intramolecular or bimolecular quadruplex, respectively [23,24]. Telomeric DNA G4s exhibit biphasic folding, generating favorable long-lived non-native intermediates through kinetic partitioning. This produces syn/anti mixed conformations and polymorphic hybrid structures, with folding timescales extending from days to weeks [25]. Moreover, investigations have revealed that chromatin condensation during cell cycle progression progressively suppresses the folding efficiency and stability of G4 structures [26]. The folding kinetics of DNA G4s are also influenced by the type of cations, with K^+^ strongly stabilizing these structures, while Li^+^ considerably weakens their stability. The folding state of DNA G4 can be affected by 5′-end sequence modifications, flanking sequence mutations (which delay G4 folding), G4-binding proteins, and deoxynucleotide triphosphate concentrations [27].

The existence and structural characteristics of rG4s are akin to those of DNA G4s; however, rG4s typically feature consecutive short GG sequences or nonuniform G-tract lengths, distinguishing them from their DNA counterparts [28]. Additionally, the presence of the 2′-OH group in RNA promotes a C3′-endo ribose pucker, influencing hydration and introducing steric constraints [29]. Consequently, rG4s predominantly adopt simpler parallel topological structures, which confer greater structural stability compared to DNA G4s, albeit this stability is contingent upon various factors, including sequence, ionic conditions, flanking regions, and experimental parameters [23,30]. Moreover, the stability of G4s substantially relies on oligonucleotide sequence, the type and concentration of stabilizing metal cations, and the number of G-quartets [29].

Regarding folding kinetics, telomeric rG4s undergo a series of rearrangements through antiparallel hairpin intermediates, cruciform structures (CSs), and parallel hairpins (PHs), ultimately forming an all-parallel, all-anti glycosidic angle structure. This folding process demonstrates single-path conformational diffusion without kinetic traps and relatively fast rates. However, the folding characteristics of rG4s can be influenced by intracellular cationic environments, where helicases may partially unwind G4 structures, disrupt their folded conformations, and reduce oligomerization capability. Additionally, the folding state is regulated by RNA-binding proteins and the molecular crowding environment [31]. For instance, during translation, ribosomes can physically unwind rG4 structures as they pass through the 5′ untranslated region (UTR), whereas during transcription, the velocity of RNA polymerase impacts rG4 folding efficiency [32].

**Fig. 2.**
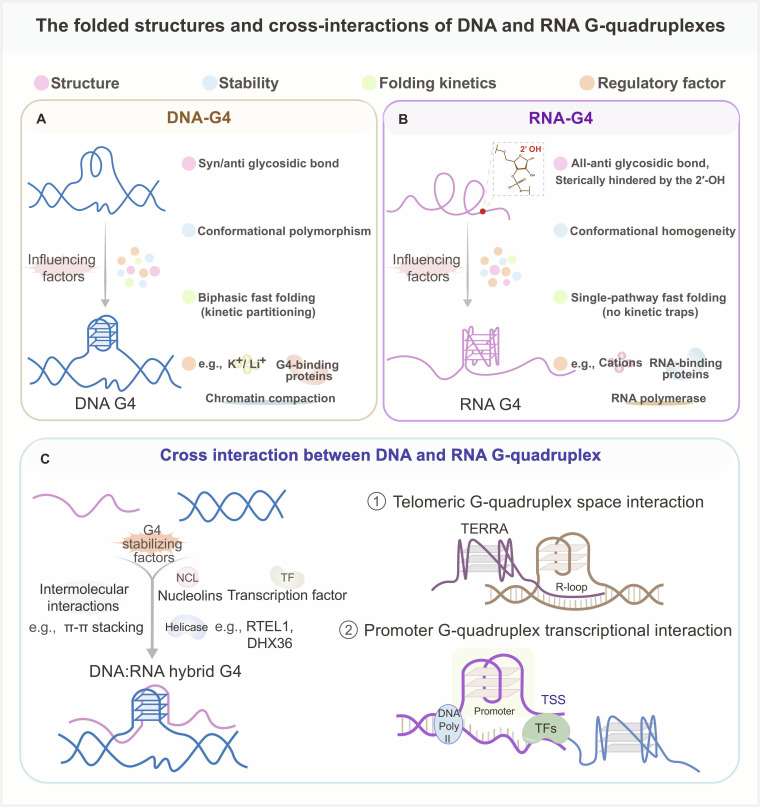
Topological folding and cross-interactions schematic of DNA and RNA G4s. (A) Folding characteristics of DNA-G4: Capable of adopting syn or anti glycosidic bond conformations; the folding process follows a biphasic rapid folding mechanism characterized by kinetic partitioning phenomena; formation and stability are influenced by factors such as cations (K^+^, Li^+^, etc.), G4-binding proteins, and chromatin condensation status. (B) Folding characteristics of rG4: The steric hindrance effect of the ribose 2′-OH group primarily promotes all-anti glycosidic bond conformations; the folding process transpires through a single-pathway rapid folding mechanism devoid of kinetic traps; regulatory factors include cations, RNA-binding proteins, and RNA polymerase, among others. (C) G4-stabilizing factors mediate DNA–RNA hybrid G4 formation. Telomeric DNA and RNA G4 forms establish spatial complexes involving R-loops. Promoter DNA G4s and their corresponding transcribed rG4s synergistically regulate Pol II binding, TF recruitment, and transcription start site (TSS) activity.

In summary, DNA and RNA G4s display notable differences in structure, stability, and folding kinetics, with both their stability and folding behavior being regulated by sequence, cations, binding proteins, and the surrounding molecular environment. G4s represent a unique class of DNA/RNA secondary structures that play important roles in cellular biology. Their distinctive structural conformations confer exceptional stability and physicochemical properties, allowing them to perform crucial functions in various biological processes (Fig. 2A and B).

### Cross-interactions between DNA and RNA G4s

The bidirectional crosstalk between DNA and RNA G4 structures represents an emerging and sophisticated field, involving various molecular mechanisms that provide critical insights into cellular homeostasis and disease pathogenesis. DNA and RNA G4s demonstrate colocalization or spatial proximity. Many gene promoters harbor G-rich regions capable of concurrently forming DNA G4 on the genomic DNA strand and rG4s on nascent transcripts [18]. This close spatial arrangement suggests potential functional interactions. The Dickerhoff team illustrated that the introduction of a single riboguanosine (rG) at the anti-position deoxyguanosine (dG) site in DNA G4 leads to the formation of a DNA–RNA hybrid structure. Their findings established that the 2′-OH group engages in a CH···O hydrogen bond with the C8H of adjacent guanines, promoting a parallel topology preference in rG4 and synergistically stabilizing the rG4 structure [33]. Both telomeric DNA (TTAGGG)n and telomeric repeat-containing RNA (TERRA) (UUAGGG)n form G4 structures that interact functionally to maintain telomere integrity [34]. G-rich regions at replication origins and intergenic loci can co-form DNA and RNA G4 structures. Furthermore, G4-DNA and G4-RNA can interact directly via complementary sequences/loops, resulting in G4-duplex hybrids that modulate the stability and function of both structures [18]. The G4 planes of both DNA and RNA G4s may undergo intermolecular interactions through stacking/intercalation (e.g., π–π stacking), influencing their folding pathways and thermodynamic stability [35]. Nucleolin (NCL), helicases (e.g., RTEL1 and DHX36), and transcription factors (TFs) act as molecular bridges between G4-DNA and G4-RNA through their dual-specific binding [18]. In cancer, dual G4 dysregulation occurs: (a) promoter G4-DNA/RNA interactions lead to oncogene overexpression, and (b) telomeric G4 defects contribute to telomere dysfunction in tumorigenesis (Fig. 2C).

This understanding of structural diversity and dynamic crosstalk between DNA and RNA G4s raises a fundamental question: How can these transient yet functionally critical structures be systematically characterized within the cellular environment? This challenge necessitates a shift from mere structural description to methodological exploration. The subsequent section offers a comprehensive overview of profiling techniques—from conventional biophysical methods to advanced sequencing and imaging platforms—that facilitate genome-wide and subcellular-resolution analysis of both DNA and RNA G4 topologies.

The crosstalk between DNA G4s and RNA G4s constitutes a multi-layered, dynamic network encompassing direct structural interactions, key protein mediators, and resultant functional coupling. This interplay is essential for gene expression regulation, maintenance of genome stability, and disease pathogenesis. Deciphering this intricate network yields mechanistic insights into disease etiology and unveils new opportunities for structure-based drug design.

## Structural Profiling of Nucleic Acid G4 Topologies

The human tumor genome contains abundant G4 motifs, particularly within telomeric repeats and transcriptional regulatory elements. However, the enrichment of tumor G4s remains challenging to observe and identify directly. Understanding the spatiotemporal dynamics of these metastable structures necessitates sophisticated detection platforms, as the physiological equilibrium between their folded and unfolded states directly impacts functional analysis. Advanced mapping techniques now facilitate genome-wide G4 landscape analysis, providing the necessary foundation for mechanistic studies and therapeutic development. This section systematically summarizes current G4 detection technologies, encompassing in vitro, cellular-level, and in vivo techniques, detailing detection principles, characteristics, advantages, limitations, and typical applications of various methods. It further compares the performance of different technologies and discusses future trends in G4 detection technologies.

### In vitro detection techniques for nucleic acid G4s

In vitro detection techniques form the basis for G4 research, primarily utilized to investigate the capacity of nucleic acid sequences to form G4s, alongside their structural characteristics and stability. Traditional mass spectrometry generates unique ion patterns indicative of G4-specific chemical features, allowing structural inferences through mass-to-charge ratio analysis. However, G4 folding is highly dependent on K^+^ and other ion concentrations, complicating accurate simulations of the tumor microenvironment [36]. X-ray crystallography can yield high-resolution 3-dimensional structures of G4s, thus enabling the study of static G4 structures and their complexes with ligands or proteins; nonetheless, it faces challenges with longer G4 sequences and is ill-suited for examining dynamic conformational changes of G4s [11]. The electrophoretic mobility shift assay (EMSA) detects the presence and topology of G4s by analyzing differences in electrophoretic mobility between G4s and other nucleic acid structures, although the complexity of G4 topologies in tumors may lead to misinterpretation of enriched structures [37]. Surface plasmon resonance (SPR) employs interactions between G4-specific binding molecules and G4 structures to quantify binding affinity and kinetic parameters for G4 identification [38]. These conventional methods enable multi-dimensional detection and characterization of G4 formation capability, structural features, and stability.

**Fig. 3.**
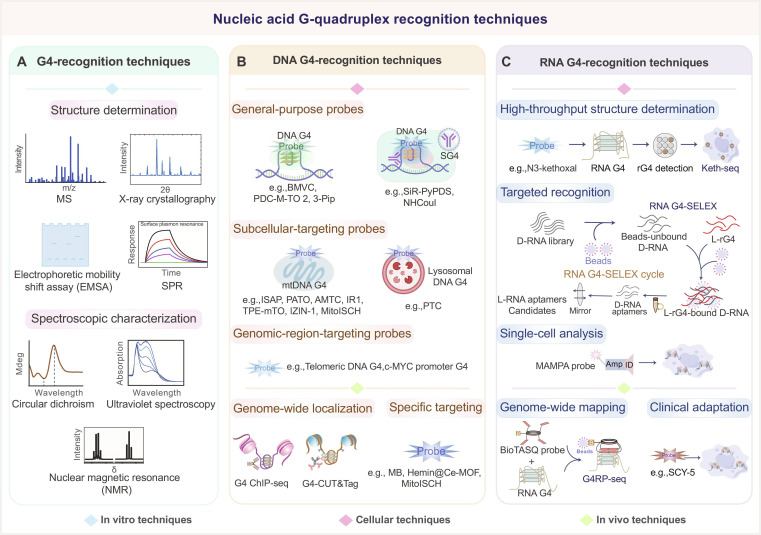
Methods for the recognition of DNA and RNA G4s. (A) In vitro universal G4 recognition technologies encompassing structural determination: mass spectrometry (MS), x-ray crystallography, electrophoretic mobility shift assay (EMSA), and surface plasmon resonance (SPR); spectroscopic characterization: circular dichroism (CD) spectroscopy, ultraviolet (UV) spectroscopy, and nuclear magnetic resonance (NMR) spectroscopy. (B) DNA G4-specific recognition technologies, including cellular-level universal probes, subcellular (mitochondrial, lysosomal) targeted probes, and genome region (telomere, c-MYC promoter) targeted probes, as well as in vivo targeting strategies through genome-wide localization techniques including G4 ChIP-seq and G4 CUT&Tag. (C) rG4-specific recognition technologies, covering high-throughput structural determination, SELEX-based targeted aptamer screening, single-cell analysis, G4RP-seq genome-wide localization, and clinical translation adaptation directions.

In addition to structural identification methods, spectroscopic approaches constitute the most commonly used in vitro detection techniques for G4s, facilitating both qualitative and quantitative analysis. Circular dichroism (CD) is the gold standard for characterizing G4 topology, exhibiting distinctive spectral features that can differentiate among parallel, antiparallel, and hybrid G4 conformations [39]. Ultraviolet spectroscopy (UV) assesses G4 stability through melting temperature (Tm values), with higher Tm values indicating greater G4 stability [39]. Nuclear magnetic resonance (NMR) spectroscopy can elucidate G4 3-dimensional structures at the atomic level, providing detailed information about hydrogen bonding, stacking interactions, and ligand binding sites; however, its large sample requirements and high experimental costs limit widespread application [40]. Additionally, small-molecule fluorescent probes, such as thiazole orange derivatives, exhibit enhanced fluorescence signals upon binding to G4s [41]. Overall, various spectroscopic detection methods provide critical support for characterizing G4 conformational stability, structural information, and abundance in tumors; nevertheless, their detection efficacy and result accuracy heavily depend on experimental conditions and are susceptible to influences from in vitro environmental factors such as ions and pH. Methodological biases arising from environmental mismatches directly impact the accurate determination of tumor G4 enrichment characteristics, while differing techniques exhibit distinct limitations and bias types (Fig. 3A).

### Cellular-level detection techniques for nucleic acid G4s

Cellular-level detection techniques are utilized to investigate the distribution, dynamic changes, and functions of G4s under physiological conditions within cells. Compared to in vitro techniques, these methods more closely mimic actual physiological environments, providing a better reflection of the true state of intracellular G4s. For instance, immunofluorescence techniques employing G4-specific antibodies (such as BG4 and SG4) can identify G4s within cells; however, these methods necessitate cell fixation and do not allow for real-time monitoring of G4s in living cells [42,43]. Fluorescent dyes such as thioflavin T (ThT), N-methyl mesoporphyrin IX (NMM) [44], NBTE [45], and N-TASQ [46] specifically bind to G4s, facilitating qualitative and quantitative detection of G4s in living cells. Furthermore, fluorescence lifetime imaging (FLIM) enables visualization of dynamic changes of G4s in living cells by detecting variations in fluorescence lifetime of G4-binding probes upon binding to G4s [47]. Within tumor cells, DNA and RNA G4s exhibit notable differences in genomic localization, dynamic regulation, and biological functions. The precise differentiation between these 2 types is crucial for elucidating the mechanisms of G4-mediated tumorigenesis. Currently, based on distinct sequence and structural preferences of probes in molecular recognition, probes can be categorized into 2 groups: those that specifically recognize DNA G4s and those that specifically recognize rG4s.

#### Cell-based specific recognition probes for DNA G4 structures

Since Gellert et al. [48] first observed in 1962 that G-rich sequences in DNA can form 4-stranded helical structures in vitro, DNA G4 has emerged as a leading focus in nucleic acid structure research. A comprehensive system for detection and structural analysis of DNA G4 has since been established, offering excellent experimental accessibility due to the high stability of DNA. Research indicates that DNA G4 fluorescent probes have progressed from basic recognition to dynamic monitoring within living cells and subcellular targeting, thereby providing advantages in analyzing the tumor physiological microenvironment that in vitro detection cannot replicate. Consequently, these probes serve as core tools for investigating the enrichment and dynamic changes of DNA G4 in tumors.

Specifically, 3,6-bis(1-methyl-4-vinylpyridinium) carbazole diiodide (BMVC), the pioneering fluorescent probe targeting DNA G4 structures, established the foundation for the design of DNA G4-specific probes [49]. Monchaud and colleagues [50] fused pyridine dicarboxamide groups with thiazole orange (TO) to create PDC-*M*-TO 2, enhancing the probe’s binding affinity for DNA G4. The SiR-PyPDS developed by the Di Antonio team [51] advanced probe application to the single-molecule level, facilitating real-time imaging of DNA G4 dynamic in living cells. The ratiometric fluorescent biosensor NHCouI, designed by Nie and colleagues [52], provided self-referenced quantification of G4 formation within cells. Cyclometalated platinum(II) complexes, such as 3-Pip, broadened the category of phosphorescent probes due to their high affinity [53]. Mitochondrial-targeting probes ISAP [54], PATO [55], AMTC [56], IR1 [57], TPE-mTO [58], IZIN-1 [59], and MitoISCH [60] enabled dynamic tracking of mitochondrial DNA (mtDNA) G4 at the subcellular level across various tumor cell lines. Probes like BEPQ-1 [61] and TOVJ [62] specifically target telomeric G4 structures, allowing precise analysis of their dynamic alterations. The PTC [63] probe transcends the traditional targeting range of cell nuclei and mitochondria, achieving localization and detection of G4-DNA within lysosomal subcellular organelles, thereby expanding the applicability of G4 probes. Probes such as coumarin-benzothiazole [64], Cy-1 [65], MRY-3 (c-MYC G4) [66], and acridine-MYB (B-MYB G4) [67] selectively target G4 structures in the promoter regions of critical oncogenes like c-MYC and B-MYB, facilitating functional studies on the interplay between oncogene transcriptional regulation and G4 structures.

This progression indicates that fluorescence signal changes due to G4 conformational transitions have emerged as a primary technical approach for the qualitative, quantitative, and dynamic tracing of DNA G4 in living cells, offering unique capabilities for in situ analysis of DNA G4 enrichment in the tumor microenvironment. Technological development and application priorities reflect an evolution from single-structure recognition to multifunctional dynamic monitoring of DNA G4 fluorescent probes. Nonetheless, core challenges persist, including nonspecific binding of fluorescent probes, quantitative inaccuracies arising from complex intracellular environments, and controversies regarding signal attribution. Current research lacks a unified evaluation system for probe specificity and anti-interference capabilities, as well as high-performance probes tailored to tumor pathological microenvironments, highlighting substantial gaps in detection technologies at the in vivo and tissue levels. Overcoming the specificity bottleneck in molecular design and addressing existing methodological biases are essential for accurately analyzing DNA G4 enrichment characteristics and biological functions in tumors (Fig. 3B and Table 1).

**Table 1.** Emerging detection probes for DNA G-quadruplex structures

Probes	Chemical structure	Wavelength λ_ex_/λ_em_ (nm)	Targets	Cell type	Ref.
ISAP	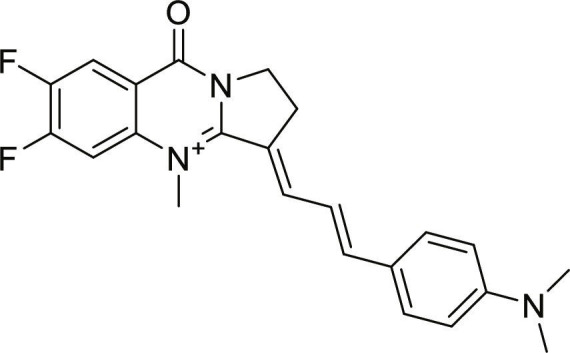	640/702	mtDNA G4	HEK293T MCF10A HepG2/A549 MCF-7 HeLa	[54]
PATO	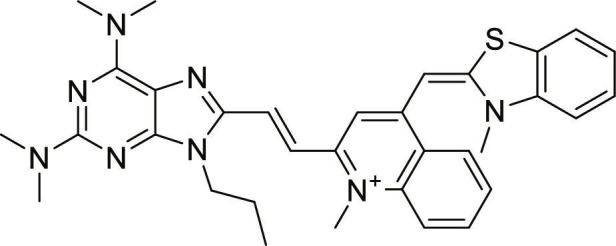	505/720	mtDNA G4	HepG2	[55]
AMTC	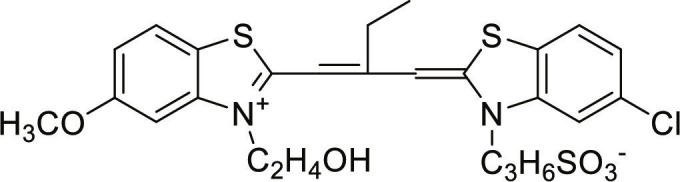	559/570–630	mtDNA G4	HeLa MCF-7	[56]
IR1	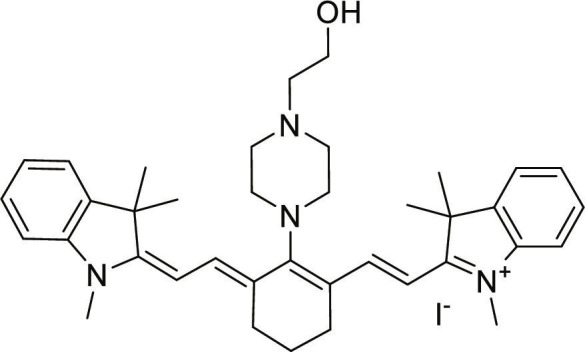	533/800	MitoG4	HCT116	[57]
TPE-mTO	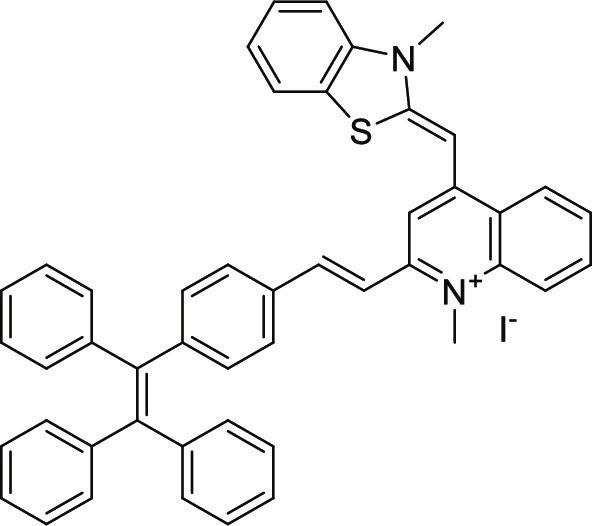	488/535	mtDNA G4	HepG2 A549 SKOV-3	[58]
IZIN-1	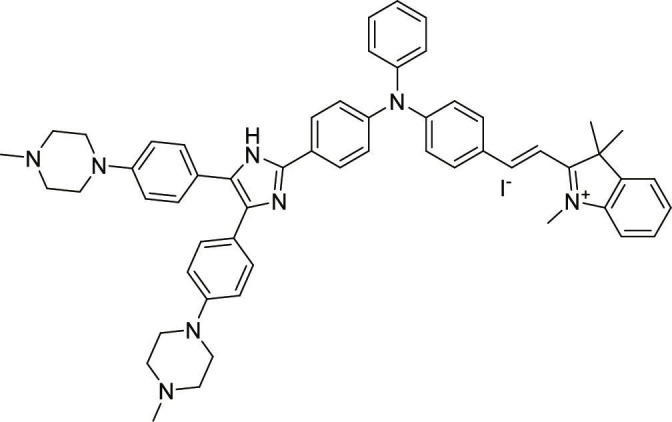	538/660	mtDNA G4	A549	[59]
MitoISCH	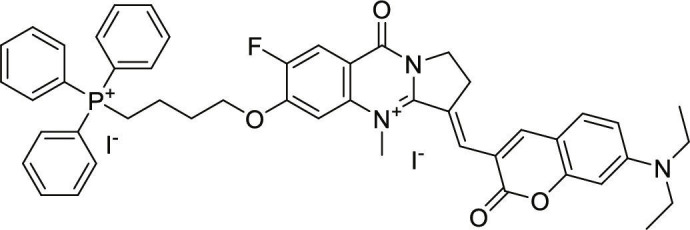	560/650	mtDNA G4	HeLa	[60]
BEPQ-1	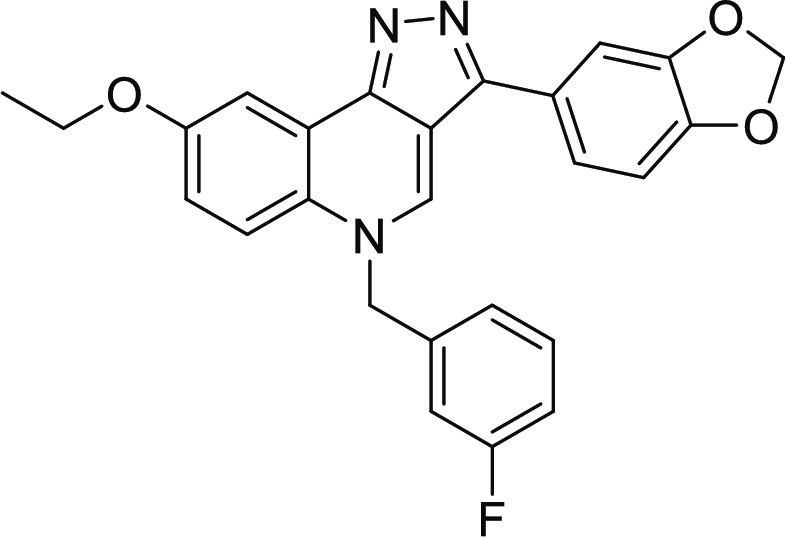	386/516	Telomeric multimeric G4	A549	[61]
TOVJ	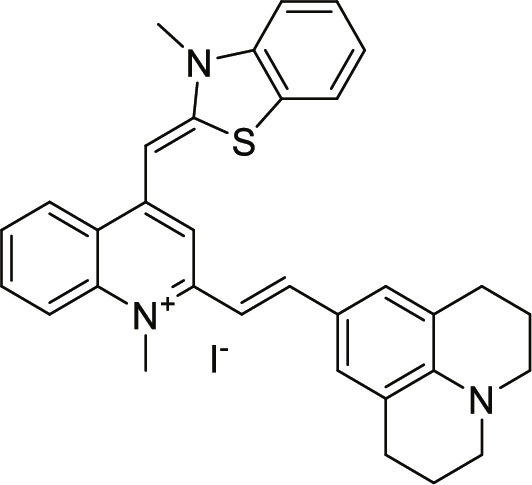	492/694	Antiparallel telomere G4-DNA	HeLa	[62]
PTC	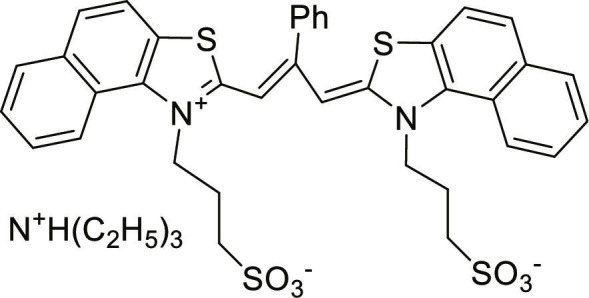	580/626	Lysosome G4-DNA	MCF-7	[63]
Coumarin-benzothiazole	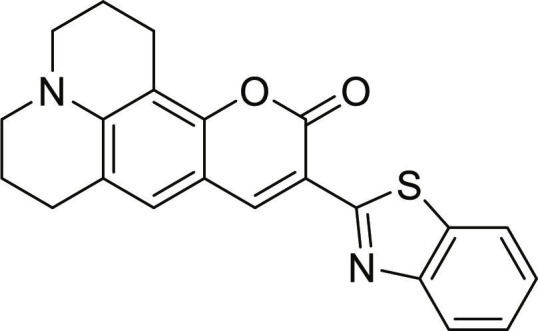	490/500–650	*c-MYC* G4-DNA	/	[64]
Cy-1	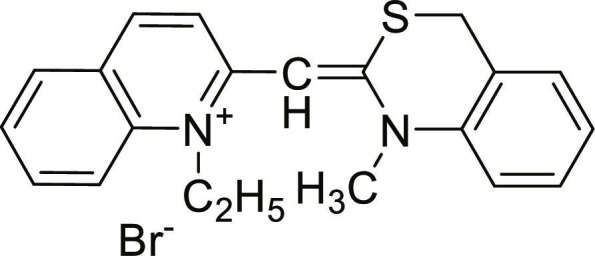	485/530	*c-MYC* G4-DNA	/	[65]
MRY - 3	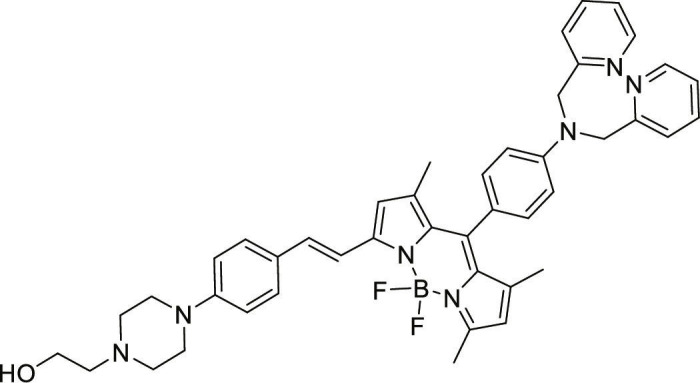	575/615	*c-MYC* G4-DNA	4T1	[66]
Acridine-MYB	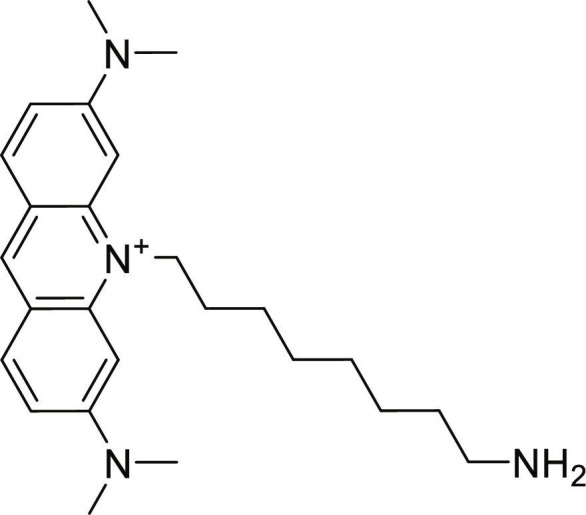	495/523	B-MYB G4-DNA	A549	[67]

#### Discriminative recognition of cellular-level rG4 structures

As a key functional secondary structure in tumor pathological processes, research on rG4 has garnered increasing attention. Nonetheless, precise detection and in situ characterization of rG4s remain hindered by various factors, including the inherent conformational dynamics of RNA molecules, susceptibility to degradation, and the complexities of in vivo environments. Recent advancements in specific probes, single-cell sequencing, live-cell imaging, and related technologies have led to breakthroughs in rG4 detection and targeting.

At the high-throughput structural identification level, Weng et al. [68] developed the N3-kethoxal probe combined with Keth-seq technology, facilitating large-scale detection of rG4s at the cellular level. The Kwok team introduced SHALiPE (selective 2′-OH acylation analyzed by primer extension), which employs differential RNA 2′-OH acylation to map rG4 structures with single-nucleotide resolution [69], together establishing a technical foundation for rG4 omics-level structural analysis. In targeting recognition strategies, the rG4-SELEX method produces L-RNA aptamers specifically directed at rG4 structures, providing a novel molecular tool[70]. Breakthroughs in single-cell analysis have been particularly important; the modular assembly multifunctional probe analysis (MAMPA) developed by Li and colleagues [71] achieved the first visualization of individual endogenous rG4 structures in human cells. These technological advancements continually expand the methodological toolbox for rG4 research, offering unprecedented tools and perspectives for understanding these complex regulatory elements (Fig. 3B and Table 2).

**Table 2.** Emerging detection probes for RNA G-quadruplex structures

Probes	Chemical structure	Wavelength λ_ex_/λ_em_ (nm)	Targets	Cell type	Ref.
TCB-1	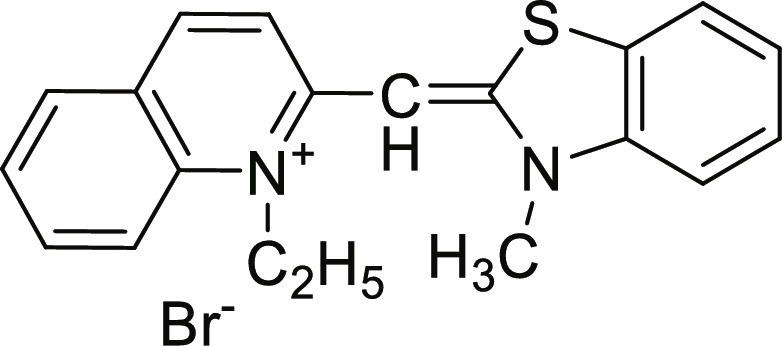	488/500–600	G4-RNA	HeLa MCF-7	[153]
SCY-5	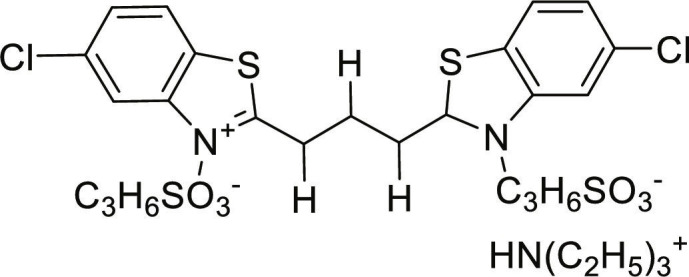	570/653	G4-RNA	HUVEC MCF-10A HeLa/A549 MCF-7 SMMC7721	[80]

In summary, the academic community has acknowledged the important biological functions of rG4s, establishing a methodological system encompassing structural mapping, targeted recognition, and single-cell visualization, which advances rG4 research into a new phase. However, the inherent instability of RNA, the complexities of intracellular microenvironments, and methodological design flaws still impede existing technologies from accurately reproducing the folding, dynamics, and functions of rG4s under pathological conditions, with issues of insufficient detection specificity. Future efforts must focus on enhancing specificity to effectively distinguish between DNA and RNA G4s, developing efficient rG4 detection methods, and establishing standardized detection systems to mitigate inherent issues such as RNA degradation. Such advancements are essential for elucidating regulatory mechanisms of rG4s in diseases like tumors and for providing a robust scientific basis for rG4-targeted disease diagnosis and treatment (Fig. 3C).

### In vivo detection techniques for nucleic acid G4s

Precise detection and in situ characterization of G4s serve as core prerequisites for elucidating their functions in processes such as tumorigenesis. In recent years, G4 detection technologies have progressed substantially from in vitro to in vivo applications, transitioning from general recognition to specific targeting. However, substantial limitations remain regarding the clinical translation potential of existing technologies, which severely constrains the understanding of the true biological functions of G4s.

#### In vivo detection techniques for DNA G4s

The development of in vivo DNA G4 detection technologies has increasingly matured. At the whole-genome localization level, G4 ChIP-seq (chromatin immunoprecipitation sequencing based on antibodies) [72] and G4 CUT&Tag (targeted cleavage and tagging) [73,74] technologies have pioneered the whole-genome localization of endogenous DNA G4s, providing evidence for the in vivo existence of DNA G4s. Nevertheless, these technologies rely heavily on the targeting accuracy of G4-specific antibodies, which commonly exhibit issues such as nonspecific binding, cross-recognition of other nucleic acid secondary structures, and low signal-to-noise ratios in high-chromatin backgrounds, raising concerns about the reliability of their results. Enhancing this approach, the LiveG4ID-seq platform developed by Teng et al. [26] integrates G4-specific labeling probes with CUT&Tag technology, substantially improving the detection sensitivity of chromatin-associated DNA G4s and enabling dynamic capture of DNA G4s in a cell cycle-dependent manner. In terms of specific targeting detection, methylene blue (MB) serves as a label-free electrochemical probe that detects telomerase activity through binding to telomeric DNA G4s, successfully applied to urine samples, marking a preliminary clinical translation of DNA G4 detection [75]. Hemin@Ce-MOF nanosensors, characterized by high sensitivity, achieve selective detection of trace telomeric DNA G4s, optimizing the detection performance further [76,77]. The development of the MitoISCH fluorescent probe addresses the need for organelle-specific detection, enabling real-time tracking of dynamic changes in mtDNA G4s and providing opportunities for elucidating the functions of DNA G4s at the subcellular level [60]. In summary, in vivo DNA G4 detection has evolved from initial localization to dynamic monitoring and clinical translation; however, methodological reliability, recognition specificity, and physiological authenticity remain to be fully verified. Future efforts should focus on establishing standardized detection and quality control systems (Fig. 3B).

#### In vivo detection techniques for rG4s

In comparison to DNA G4s, in vivo rG4 detection technologies remain limited in their capacity for dynamic monitoring and clinical applications, and their technical frameworks require further enhancement. rG4 sequencing (rG4-seq) integrates reverse transcriptase stalling with high-throughput sequencing, marking the first comprehensive demonstration of rG4 structures within the human transcriptome [78]. However, this technology relies on in vitro RNA extraction and processing, complicating the preservation of the natural intracellular microenvironment. G4-RNA-specific precipitation sequencing (G4RP-seq) employs G4-specific probes (BioTASQ) for affinity capture combined with sequencing, achieving whole-genome mapping of rG4s [79]. While this approach improves detection specificity to some degree, it does not fully address existing limitations. On the clinical adaptation level, the Sun team developed SCY-5, an anionic cyanine-based supramolecular probe that enables in situ rG4 imaging and quantitative analysis of clinical blood samples [80]. Overall, in vivo rG4 detection technologies remain in the exploratory stage, lacking dynamic monitoring, subcellular targeting, and robust clinical adaptation technologies, highlighting a marked gap compared to the detection capabilities for DNA G4s (Fig. 3C).

The comprehensive structural analysis techniques discussed above highlight the remarkable diversity and dynamics of G4s, providing a methodological foundation for increasingly precise exploration of their presence and conformational preferences. With these sophisticated detection platforms, researchers can progress beyond mere structural characterization to investigate the functional implications of G4 formation within complex cellular environments. This transition from structure to function is particularly critical in the context of cancer, as G4s emerge as key regulatory factors influencing genomic stability and gene expression. Consequently, the following sections will examine the multifaceted roles of G4s as integral components of oncogenic mechanisms and transcriptional control, illustrating how these noncanonical nucleic acid structures facilitate tumorigenesis and represent promising therapeutic targets.

## G4s: Pivotal Regulators in Oncogenic Mechanisms and Transcriptional Control

### G4s in cancer-associated chromosomal inheritance mechanisms

**Fig. 4.**
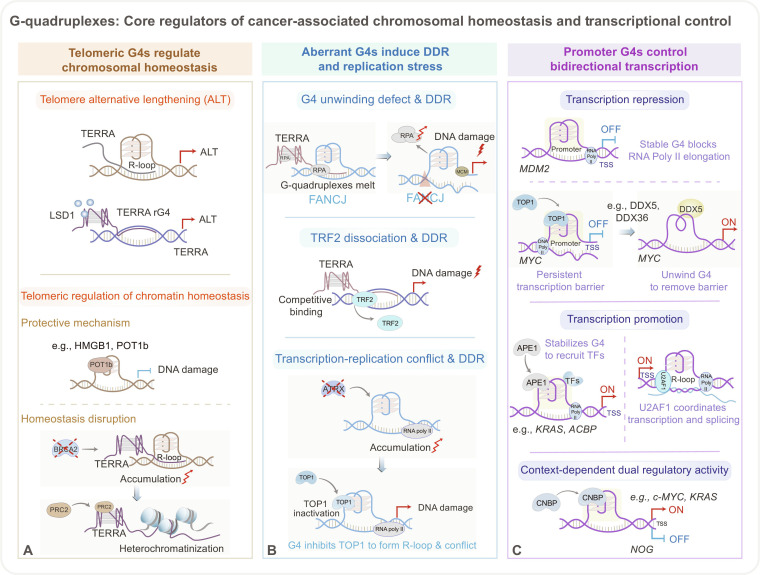
Regulatory roles of G4s in cancer-related chromosomal inheritance mechanisms. (A) Telomeric G4s in chromosome maintenance: TERRA R-loops/rG4s promote ALT, with LSD1 modulating this process; POT1b mitigates DNA damage; BRCA2 loss disrupts homeostasis via R-loops, while TERRA rG4 recruits PRC2 for heterochromatinization. (B) Telomeric G4s in DNA damage repair: FANCJ deficiency causes RPA/G4 accumulation and damage; TERRA rG4 displaces TRF2, activating DDR; ATRX loss or TOP1 inhibition triggers R-loops and replication conflicts. (C) DNA G4-mediated transcriptional regulation: G4s repress *MDM2* and *MYC*; helicases unwind *MYC* G4 for activation; APE1 stabilizes *KRAS/ACBP* G4s to recruit TFs; CNBP activates *c-MYC/KRAS* but represses *NOG*.

G4s may play diverse roles in chromosome-associated genetic mechanisms, primarily impacting chromosome stability, gene expression, and telomere maintenance, thereby contributing to cancer development. Increasing evidence suggests that chromosomal G4 structures are involved in telomere homeostasis, which directly influences tumor progression. For example, in human osteosarcoma cells, TERRA-mediated R-loops facilitate the formation of telomeric DNA G4 structures while enhancing alternative lengthening of telomeres (ALT) activity [81]. Furthermore, G4 structures within TERRA interact with lysine-specific demethylase 1A (LSD1) to promote ALT-dependent telomere maintenance in osteosarcoma cells [82]. This interaction demonstrates how noncanonical nucleic acid structures (G4s and R-loops) collaborate with epigenetic modifiers to regulate telomere maintenance programs. This microenvironment specificity is also evident in the considerable interference of G4 with chromatin state. In HeLa cells, the stability of telomeric G4s correlates with BRCA2 expression. Depletion of BRCA2 results in TERRA-R-loop accumulation, stabilizing telomeric G4s and recruiting polycomb repressive complex 2 (PRC2), ultimately leading to trimethylation of histone H3 at lysine 27 (H3K27me3) and subsequent chromatin condensation at telomeres [83]. Additionally, high-mobility group box 1 protein (HMGB1) [84] and protection of telomeres 1b (POT1b) [85], encoded by the *POT1* gene, inhibit telomeric DNA damage by recognizing or unwinding telomeric DNA G4, forming the core of the telomere end protection mechanism. Collectively, these findings illustrate that G4 structures are essential for maintaining chromosomal integrity and preventing DNA damage, ultimately promoting tumorigenesis. However, the mechanisms by which they operate are highly dependent on telomere-specific proteins, making general extrapolation to the entire genome challenging (Fig. 4A).

Emerging evidence indicates that chromosomal G4s can activate DNA damage response (DDR) pathways, markedly influencing cancer development. At telomeres, human replication protein A (RPA) binds to and resolves G4 structures [86], while in multiple myeloma (MM), TERRA G4 stabilization promotes the dissociation of telomeric repeat-binding factor 2 (TRF2), thereby triggering DDR activation [87]. Moreover, G4-mediated epigenetic remodeling demonstrates high site specificity. During X chromosome inactivation, the G4 structure of X-inactive specific transcript (Xist) RNA interacts with PRC2 to inhibit H3K27me3 activity [88]. In contrast, the G4 in the hTERT promoter binds to TRF2, recruiting the EZH2/PRC2 complex to induce H3K27 trimethylation, thus repressing hTERT expression in glioblastoma (GBM) [89]. Concurrently, G4-induced genomic instability largely results from its physical barrier properties, disrupting the coordination between transcription and replication machineries. Chromosomal DNA G4s contribute to genomic instability by inhibiting human DNA topoisomerase I (TOP1) activity during transcription in HCT116, MDA-MB-231, and HeLa cells, leading to the accumulation of torsional stress, increased R-loop (RNA–DNA hybrid) formation, and ultimately resulting in transcription-replication conflicts, DNA double-strand breaks, and genomic instability [90]. Similarly, defects in the alpha thalassemia/mental retardia syndrome x-linked (ATRX) [91] or Fanconi anemia complementation group J (FANCJ) helicase [86] stabilize DNA G4 structures, inducing replication stress, DNA damage, and subsequent apoptosis in cancer cell. Recent studies have demonstrated that TERRA G4s can be pharmacologically modulated to increase the levels of telomeric DNA:RNA hybrids, thereby inducing telomere damage and activating the ALT pathway via the RNaseH1 regulatory mechanism [92]. Additionally, these structures can be utilized for selective RIBOTAC-mediated degradation of TERRA [93]. These findings indicate that G4 structures exhibit dual protective and destructive potential at the chromosomal level, with the resulting phenotype contingent upon cell type, genetic background, and the dynamic balance of associated helicases and binding proteins (Fig. 4B).

### DNA G4s: Key regulators of transcriptional modulation

DNA G4s are extensively enriched near transcription start sites and are recognized as cis-regulatory elements that bidirectionally modulate gene expression in a context-dependent manner. Traditionally, G4 structures located in G-rich promoter regions are viewed as transcriptional repressors. For instance, in liposarcoma, the stabilization of a G4 conformation within the MDM2 promoter P2 region directly obstructs RNA polymerase II elongation and suppresses p53 degradation by silencing MDM2 transcription and subsequently apoptosis in cancer cells [94]. However, this repressive effect is not static but exists in a dynamic equilibrium influenced by specific helicases. On the one hand, G4 formation in the MYC promoter, aided by TOP1, creates a transcriptional barrier [95]. Conversely, DEAD-box helicases (such as DDX5) or DExH-box helicases (such as DHX36) can unwind G4 structures through their high binding affinity, thus restoring transcriptional activity [96,97]. Collectively, these findings position DNA G4 as a dynamic regulatory switch, enabling cells to achieve rapid transcriptional responses to growth signals by modulating helicase recruitment without altering the underlying DNA sequence.

In addition to their repressive functions, G4 structures facilitate transcription by acting as scaffolds for the binding of specific TFs. In pancreatic cancer, AP endonuclease 1 (APE1) binds to and stabilizes G4 formation in the Kirsten rat sarcoma viral oncogene homolog (KRAS) promoter region, establishing a stable platform for the recruitment of TFs such as MYC-associated zinc finger protein (MAZ) and poly (adenosine diphosphate-ribose) polymerase 1 (PARP1), which collectively drive KRAS oncogene expression [98]. Similarly, the G4 structure in the acyl-CoA-binding protein (ACBP) gene promoter acts as a transcriptional enhancer in HepG2 cells by facilitating the binding of regulatory TFs [99].

Moreover, the functional scope of G4 is expanding. In HEK293T cells, U2 small nuclear RNA auxiliary factor 1 (U2AF1) simultaneously binds to both R-loops and DNA G4 structures, competitively occupying 3′ splice sites to coordinate transcription initiation and mRNA splicing processes [100]. This dual binding capability ensures that genes with complex transcriptional regulation—comprising both G4s and R-loops—are aligned with the appropriate RNA processing pathways, thus maintaining fidelity in gene expression.

G4 structures exhibit context-dependent dual functionality in gene transcription regulation, exemplified by the cellular nucleic acid-binding protein (CNBP), which differentially regulates transcriptional outcomes through G4 unfolding in promoter regions. Specifically, CNBP activates oncogenes c-MYC and KRAS in HeLa cells while concurrently suppressing NOGGIN (NOG) gene expression [101]. This context dependency suggests that G4s are not inherently activating or repressive; instead, their functional outcomes are dictated by the local promoter architecture and the specific proteins that interact with them (Fig. 4C). Overall, as dynamic switches within the cancer transcriptional regulatory network, DNA G4 structures encompass multiple roles, including serving as physical barriers and scaffolds for protein recruitment. Nonetheless, their functional outputs display substantial nondeterminacy, complicating precise predictions regarding the functional trajectory of G4 at specific genomic sites. Future research should aim to decipher the regulatory code of G4s under varying conditions, leveraging this environment specificity to develop more tailored anticancer therapeutic strategies.

In conclusion, DNA G4s demonstrate dual properties as both “physical barriers” and “recruitment scaffolds” in chromosomal homeostasis and transcriptional control, with their functional outputs strictly dependent on subcellular localization and interacting protein partners. This high degree of environmental specificity constitutes a dynamic regulatory network at the DNA level. Moreover, the regulatory landscape surrounding G4s extends beyond transcription to encompass post-transcriptional biological activities, where rG4s, characterized by their unique single-stranded flexibility and spatial conformations, influence cancer progression in more complex dimensions.

## rG4s: A Multidimensional Functional Network Regulating the Entire Landscape of Gene Expression

rG4s are increasingly recognized not merely as noncoding secondary structures but as dynamic regulatory molecular switches. Research has demonstrated that rG4s, with their unique thermodynamic stability and conformational plasticity, establish a multidimensional regulatory network that links RNA stability control, precise translational guidance, and functional organization within the nucleus.

### The presence of rG4 structures affects RNA stability

Characterized by minimal structural dynamics, rG4s adopt thermodynamically stable conformations, typically manifesting as compact, closed architectures. Numerous studies have identified rG4 structures as critical regulators of mRNA stability through various mechanisms. Firstly, rG4 exhibits marked environmental sensitivity, allowing them to function as a “sensors” for metabolic stress in tumors. In MCF7 breast cancer cells, oxidative stress-induced generation of reactive oxygen species (ROS) leads to the oxidation of guanine bases within the Flap endonuclease 1 (FEN1) 5′UTR rG4 structure, resulting in its destabilization and a subsequent reduction in FEN1 mRNA stability. The interaction between heterogeneous nuclear ribonucleoprotein A1 (hnRNPA1) and FEN1 rG4 inhibits γH2AX expression and modulates the DDR [102]. This redox–rG4 axis highlights a previously underappreciated mechanism by which metabolic stress alters gene expression programs in tumor cells. Secondly, the regulation of rG4 stability is deeply reliant on the synergistic interaction and dynamic resolution facilitated by specific proteins. For instance, the helicase DHX36 modulates mRNA turnover in HEK293 cells by resolving rG4 structures in 3′UTRs [103]. This targeted resolution acts as a positive regulator of transcript stability for key transcripts—including amyloid precursor protein (*APP*), anterior pharynx defective 1A (*APH1A*), histone cell cycle regulator defective homolog A (*HIRA*), and lamin B1 (*LMNB1*) in U2OS osteosarcoma cells [104]. Conversely, the stress granule (SG) core protein GTPase-activating protein (SH3 domain)-binding protein 1 (G3BP1) directly binds rG4 structures in HeLa and HEK293T cells, enhancing the stability of mRNAs for paired-like homeodomain transcription factor 1 (*PITX1*), KH-type splicing regulatory protein (*KHSRP*), and ARP2 actin-related protein 2 (*ACTR2*) [105]. This interaction connects rG4 recognition to stress-induced translational reprogramming by sequestering transcripts into SGs.

**Fig. 5.**
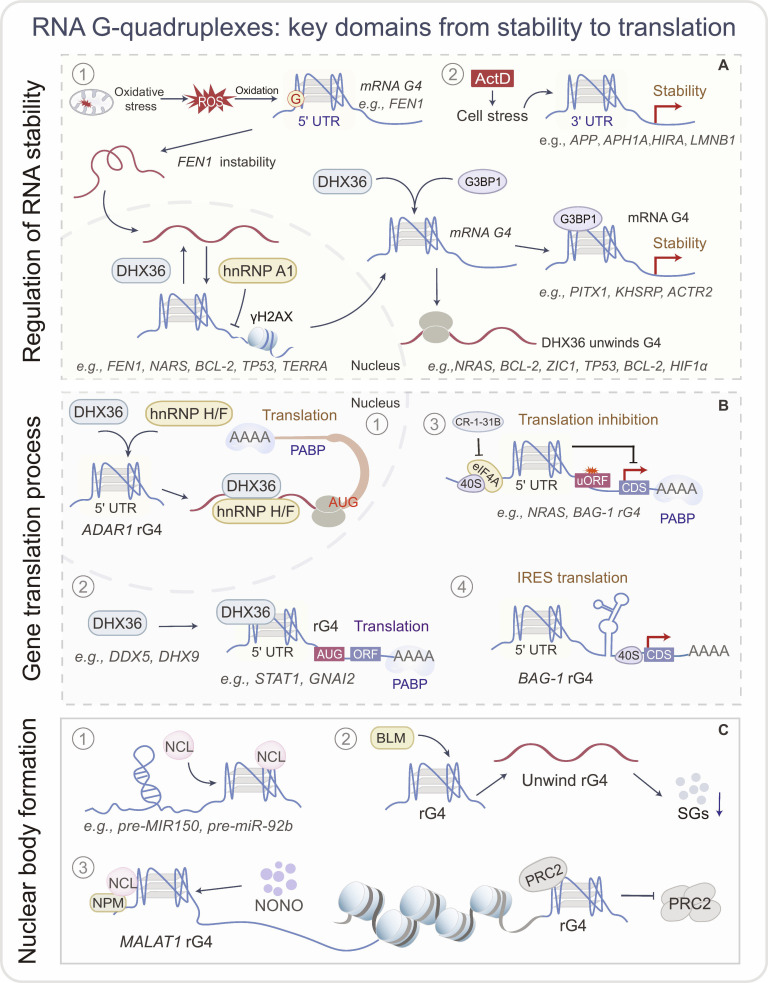
rG4s: Key structural domains from stability to translation. (A) rG4s modulate RNA stability. ① Under oxidative stress, ROS disrupts the FEN1 5′UTR G4, leading to reduced stability. ② ActD-induced stress promotes rG4 folding in the 3′UTR of *APP*, *APH1A*, *HIRA*, and *LMNB1* mRNAs, enhancing their stability. ③ DHX36 unwinds rG4s in NARS/BCL-2 mRNAs. G3BP1 directly binds to rG4s, positively regulating the stability of *PITX1*, *KHSRP*, and *ACTR2* mRNAs. (B) rG4s regulate gene translation processes. ① The ADAR1 5′UTR rG4 binds DHX36 and cooperates with hnRNP H/F to regulate translation. ② Helicases DHX36, DHX9, and DDX5 unwind 5′UTR rG4s in STAT1 and GNAI2 mRNAs. ③ The eIF4A helicase inhibitor CR-1-31B disrupts the *KRAS* rG4, while BAG-1, in coordination with uORF, represses translation. ④ The rG4 in *BAG-1* mRNA maintains IRES activity to initiate translation. (C) rG4s recruit proteins to regulate nuclear-related functions. ① rG4s in pre-miRNAs (such as pre-MIR150 and pre-miR-92b) are recognized by NCL. ② BLM unwinds rG4 structures, thereby suppressing SGs formation. ③ The MALAT1 rG4 interacts with NCL, NPM, and NONO to promote nuclear body assembly and recruits PRC2 to inhibit nucleosome function.

Collectively, these findings establish that rG4s exert precise control over mRNA stability through mechanisms involving de novo structural folding, targeted structural disruption, and protein-mediated stabilization. These regulatory processes substantially influence cancer progression by modulating the expression of key oncogenes and tumor suppressors. Pharmacological modulation of rG4 stability presents opportunities to selectively reprogram mRNA stability in cancer cells (Fig. 5A).

### rG4: A molecular switch for dynamic regulation of translation program

The regulatory role of rG4 in gene expression transcends static paradigms, functioning as a dynamic element that integrates helicase activity, RNA-binding proteins, and cellular signals to fine-tune protein synthesis at the post-transcriptional level. Research conducted in 2017 highlighted the significance of rG4s in modulating gene translation. Specifically, the translation of oncogenic mRNAs critically relies on the dynamic resolution of rG4 structures within the 5′UTR. rG4s located in the 5′UTR stably impedes the scanning of the 43*S* pre-initiation complex (PIC) through physical steric hindrance while also marking inhibitory upstream open reading frames (uORFs) that block the translation of downstream coding sequences (CDSs) [106]. For instance, rG4 structures formed in the 5′UTR of the proto-oncogene neuroblastoma RAS viral oncogene homolog (NRAS) mRNA exhibit translational repression [107]. This mechanism presents an opportunity for pharmacological intervention, and the eukaryotic initiation factor 4A (eIF4A) helicase inhibitor CR-1-31B disrupts rG4 structures, thereby regulating *KRAS* mRNA translation and interfering with oncogenic signal transduction [108].

Helicase-mediated rG4 remodeling is essential for cancer translational reprogramming, wherein the functional role of rG4 is contingent upon its dynamic resolution by helicases. For example, the helicase DHX36 interacts with the rG4 in the 5′UTR of adenosine deaminase acting on RNA 1 (ADAR1) mRNA to regulate ADAR1 translation [109]. In GBM, DHX36 collaborates with translation regulators, such as heterogeneous nuclear ribonucleoproteins H/F/H1/H2 (hnRNP H/F), to drive rG4-mediated translational reprogramming, a synergistic effect directly linked to tumor therapy resistance [110]. Similarly, in hepatocellular carcinoma (HCC), the RNA helicase DDX5 resolves rG4 structures in the 5′UTR of signal transducer and activator of transcription 1 (STAT1) mRNA, modulating STAT1 translation [111]. These observations indicate that rG4 has emerged as a target site for helicase action, thereby enabling selective regulation of cellular fate by altering RNA conformations.

Furthermore, rG4 serves a unique “dual-functional scaffold” role in switching translation modes. Beyond cap-dependent translation, rG4 structures play a regulatory role in cap-independent translation. In colorectal cancer, the rG4 structure within the 5′UTR of the anti-apoptotic Bcl2-associated athanogene 1 (*BAG-1*) employs a dual regulatory mechanism. It collaborates with inhibitory uORFs to hinder ribosomal scanning and translation of in-frame isoforms, thereby suppressing cap-dependent translation; simultaneously, rG4 acts as an architectural scaffold that maintains the global secondary structure of 5′UTR, facilitating internal ribosome entry site (IRES)-driven cap-independent translation [112]. This capability allows cancer cells to rapidly transition from canonical translation to anti-apoptotic programs under stress conditions (Fig. 5B).

In summary, the essence of rG4 at the translational level lies in its high conformational plasticity and multidimensionality functionality. It serves not only as a negative regulator of protein synthesis but also a central coordinator for driving the reprogramming of cancer-specific translation programs. Therefore, targeting the dynamic equilibrium of rG4—by either stabilizing its inhibitory conformation or disrupting its interaction with helicases—represents a novel strategy for reshaping the cancer translational landscape within precision medicine.

### rG4s regulate nuclear-associated functions through protein recruitment

The functional repertoire of rG4s has expanded beyond cytoplasmic translation to encompass the nuclear interior, establishing them as core regulatory units modulating nuclear spatial architecture and biochemical reactions. rG4s are not merely carriers of genetic information; they serve as essential structural scaffolds that, through multidimensional protein recruitment and allosteric regulation, are deeply involved in nuclear body assembly, chromatin modification, and noncoding RNA processing. Specifically, rG4s act as anchors for assembling subnuclear structures. For example, the G4 structure within the long noncoding RNA metastasis-associated lung adenocarcinoma transcript 1 (MALAT1) has been shown to effectively recruit proteins such as NCL, nucleophosmin (NPM) [113], and non-POU domain-containing octamer-binding protein (NONO) [114]. This recruitment promotes the targeted enrichment of these key proteins into nuclear speckles and nuclear bodies, thereby elucidating how noncoding RNAs guide protein localization and maintain the spatial order of nuclear compartmentalization.

The regulatory capacity of rG4s extends beyond simple protein sequestration to include allosteric modulation and orchestration of higher-order nuclear architecture. Importantly, rG4s function as structural decoys; for instance, they can bind directly to the catalytic core of PRC2, thereby antagonizing its interaction with nucleosome substrates and inhibiting its potentially harmful recruitment in cancer cells [115]. Moreover, Cruz and colleagues identified G4-forming sequences in human precursor MIR150 (*pre-MIR150*) and precursor microRNA-92b (*pre-miR-92b*), which have the capacity to interact with NCL [116,117], highlighting their fine-tuning role in the biogenesis of small RNAs. Additionally, Bloom’s syndrome protein (BLM), a well-characterized nuclear helicase, is capable of unwinding rG4 structures. This helicase activity is essential for its enrichment into SGs, where it serves as a negative regulator of SG assembly [118]. In summary, rG4s play vital roles in regulating nuclear functions through multiple mechanisms, including binding to nuclear proteins, interacting with nucleosome substrates, and recognizing nucleolar proteins (Fig. 5C). The multifunctionality of rG4s within the nucleus reflects a functional transition from mere sequence recognition to serving as higher-order structural scaffolds. By facilitating physical interactions with core nuclear proteins, rG4s effectively couple the local conformation of RNA with the spatial organization within the cell nucleus.

rG4s establish a multidimensional regulatory network that encompasses stability, translation efficiency, and nuclear spatial organization during cellular processes. Dysregulation of this balance in diseases such as cancer highlights the strategic role of this noncanonical RNA structure in pathophysiological mechanisms. This central function positions G4s as promising therapeutic targets. Precise intervention in the conformational balance of rG4s through pharmacological approaches—beyond simple inhibition or activation—represents a important opportunity for future advancements in biomedicine.

## Clinical Implications of Targeting G4 Conformations

Ligands targeting G4s have emerged as a highly promising class of structure-specific drugs in cancer therapy. Given the widespread distribution of G4-forming sequences in telomeres and oncogene promoters, these ligands exert their effects by interfering with genomic stability, transcriptional regulation, and replication dynamics. However, despite rapid advancements, the field remains fragmented due to chemical diversity, target heterogeneity, and an incomplete mechanistic understanding. A systematic classification based on parent chemical scaffolds provides a clearer framework for evaluating structure–activity relationships and therapeutic potential.

### Compounds targeting DNA G4 inhibit tumor progression

Reported DNA G4-targeting compounds can be classified into 5 major categories based on variations in parent core structures, each exhibiting substantial hierarchical differences concerning target specificity, mechanisms of action, and clinical translation potential.

#### Metal-based ligands

Recent studies indicate that numerous metal ligands interact with DNA G4s with high affinity and selectivity [119]. Among platinum-based ligands, Tetra-Pt(bpy) simultaneously targets the telomeric G4 and the G4 within the PTK2 (FAK) gene, inducing tumor cell senescence and inhibiting proliferation [120]. Pt-phen [121] and L1-transpt [122] specifically bind to the G4 in the MYC promoter, significantly down-regulating MYC expression and inhibiting breast cancer cell proliferation. Heteronuclear metal ligand chiral Ru^II^-Pt^II^ complexes exhibit potent G4-stabilizing activity. When encapsulated in biotin-functionalized DNA nanocages, they improve subcellular localization and cancer cell selectivity, demonstrating marked therapeutic effects against cisplatin-resistant tumors [123]. Other metal ligands, such as Br-substituted RG260 [36], specifically bind to telomeric G4, reducing telomerase-mediated telomere synthesis. The primary advantage of this class of compounds lies in their high G4-binding affinity; however, they typically suffer from off-target binding to double-stranded DNA. Additionally, the potential nephrotoxicity and neurotoxicity of metal ions severely limit their clinical applicability and dosage.

#### Quinoline/acridine-based ligands

Quinoline and acridine-based compounds exhibit high selectivity for G4s in oncogene promoters and represent one of the most extensively studied classes of G4 ligands. Quinoline ligands such as RG260 [36] and BMPQ-1 [124] selectively stabilize telomeric G4, inducing DDR, cell cycle arrest, and apoptosis at telomeres. QN-1 [125] specifically binds to the G4 in the c-MYC promoter, down-regulating MYC expression and inhibiting the proliferation of triple-negative breast cancer (TNBC) cells; its derivative 5 [126] further achieves dual targeting of topoisomerase 1 (Topo1) and c-MYC G4, exhibiting enhanced antitumor activity. Meanwhile, BMVC-8C3O stabilizes c-FOS G4 to inhibit its expression and sensitizes osimertinib-resistant lung cancer to therapy [127]. The Dudek team designed the fluorinated azobenzene switch Q-Azo4F-C, which enables reversible regulation of G4 structures in lung cancer cells via visible-light-driven conformational changes [128]. Similarly, Ma et al. [129] reported that compound 13d induces G4 formation, leading to apoptosis in gastric cancer cells. MY-8 [130] highly selectively stabilizes the G4 in the MYCN promoter, significantly down-regulating MYCN oncogene expression and inhibiting neuroblastoma proliferation. Acridine-based ligands, such as compound 3 and BRACO-19 [131], inhibit telomerase activity by stabilizing telomeric G4 formation, inducing senescence or cell death in various cancer types. While these compounds demonstrate high selectivity for G4s in oncogene promoters and some have progressed to preclinical evaluation stages, further improvements are needed in their ability to distinguish between G4s with different topologies, and off-target effects require systematic assessment.

#### Naphthalene diimide and its derivatives

The naphthalene diimide (NDI) core exhibits excellent planarity and modifiability, making it one of the most widely used cores in G4 ligand design. β-Thiomaltosyl-NDI-NMe2 can simultaneously target G4s in the KRAS and MYC promoters, down-regulating the expression of both oncogenes and inhibiting colorectal cancer cell proliferation [132]; naphthalene diimide-benzotriazole conjugates (3c) stabilize G4, down-regulate BCL-2 expression, and promote apoptosis in lung cancer cells [133]. The benzothiophene-dibenzoyl imide derivative 7C selectively stabilizes mitochondrial G4 (mtG4), disrupts mtDNA replication and transcription, and activates multiple cell death pathways [134]. The core of this class of compounds allows for functional modification, enabling the regulation of target specificity and subcellular localization through different side chains; however, some derivatives exhibit poor water solubility and insufficient cellular permeability and are cleared by hepatic metabolism, leading to extremely low bioavailability.

#### Porphyrin derivatives

The strong π–π stacking interactions between the planar aromatic macrocyclic structures of porphyrins and G-quartets make them one of the earliest and most extensively studied G4 ligands. TMPyP4 [131] stabilizes G4 structures to disrupt telomere maintenance mechanisms, affecting telomerase activity or the ALT pathway [135]. The porphyrin–guanine conjugate mPG specifically stabilizes G4 in the promoter of platelet-derived growth factor receptor β (PDGFR-β), modulating transcription in osteosarcoma [136]. While compounds in this class possess strong G4-binding affinity, their limited selectivity remains a substantial drawback.

#### Coumarin derivatives

Coumarins represent a nature-inspired heterocyclic scaffold, with substituents that can be modulated to enhance biocompatibility and selectivity. Suseela et al. [137] demonstrated that the coumarin-based compound TGP18 binds to BCL-2 G4, inhibiting transcription and inducing apoptosis through DNA damage and oxidative stress cascades. The natural coumarin-based compound COP, identified by Xiao et al. [138], stabilizes ATF4 G4, obstructs its interaction with TFAP2A, and reduces ATF4 levels, leading to cancer cell death. Currently, its efficacy and pharmacokinetic studies are still in the early stages in vivo.

#### Other heterocyclic ligands

Benzothiazole-based ligand CX-5461 [139] is a promising G4-targeting small molecule that has progressed to phase II clinical trials (NCT02103861) for the treatment of BRCA-mutated ovarian cancer and TNBC, representing a highly promising research direction. Dey et al. [140] identified anthraquinone derivatives N-1P/N-2P that interact with human telomeric G4, inducing apoptosis in breast cancer cells. Monohydrazone derivative 15 [141] stabilizes the Tel23 G4 and R-loop, inducing DDR and cell death. Dimeric aryl imidazole DIZ-3 [142] selectively stabilizes telomeric multimeric G4, inducing telomeric DNA damage and cell cycle arrest. These compounds exhibit high binding affinity but often exhibit off-target DNA interactions and cytotoxicity.

#### Oligonucleotide/hybridization-based G4 targeting strategies

Additionally, based on nucleic acid and hybrid targeting systems, Valiuska et al. [143] designed poly-purine reverse Hoogsteen hairpin (PPRH) oligonucleotides targeting G4 structures in the MYC promoter and intronic regions. Jiang et al. [144] developed a novel histone deacetylase (HDAC)/G4 dual-target inhibitor by conjugating an HDAC inhibitory moiety with an indirubin scaffold targeting G4, which displayed enhanced antitumor activity against TNBC through synergistic effects. G4 ligands have now evolved beyond a single-target model and moved toward multimodal precision therapy.

#### Peptide-based G4-targeting strategies

G4-targeting peptides represent an emerging approach aimed at overcoming the limited selectivity issues associated with small molecule ligands by leveraging protein-inspired recognition motifs. The RHAU18 peptide, a short peptide derived from an engineered RHAU (also known as DHX36)-specific motif (RSM), selectively binds to G4 structures and can disrupt transcriptional elongation or RNA translation [145]. Optimized mutants of Rap1 protein Myb1 domain-derived peptides, Myb A400 and Myb A412, exhibit strong G4-binding affinity; when conjugated with cell-penetrating peptides (CPPs) such as R₇W, they induce telomeric DNA damage in tumor cells [146]. These protein-derived G4-targeting peptides offer unique advantages in addressing selectivity issues but still face challenges in tumor selectivity, stability, and delivery, limiting their therapeutic translation.

#### G4 theranostics and drug repurposing solutions

In terms of G4-targeted delivery and theranostic platforms, Wang et al. [147] developed ApG4/LDCs, a tumor-specific theranostic platform that combines G4-stabilizing DNA carriers with stimuli-responsive ligand–drug conjugates (LDCs). This system utilizes a bifunctional fluorescent ligand (ThP-CyS) to enhance G4 binding and nuclease resistance while co-delivering sodium nitroprusside and doxorubicin. By employing tumor microenvironment-triggered factors such as glutathione and nicotinamideadenine dinucleotide phosphate oxidase (NOX), it achieves controlled drug release, real-time monitoring, and reduction of off-target effects, addressing key bottlenecks in the efficiency and specificity of G4-targeted therapies.

Drug repurposing involves utilizing approved or clinically tested compounds to target G4 structures, providing a rapid and lower-risk pathway for therapeutic development. Through ligand-based virtual screening and biophysical methods, several promising compounds—including azelastine, belotecan, and irinotecan—have been identified as effective G4 binders with notable antiproliferative effects in breast cancer cell lines [148]. However, existing drugs encounter challenges such as limited G4 selectivity, insufficient understanding of in vivo G4 targeting, and the risks of off-target toxicity. Future efforts should focus on combining drug repurposing with rational design, structural biology, and targeted delivery systems to fully exploit the therapeutic potential of G4 targeting.

In summary, compounds targeting G4 structures can be classified based on their parent cores into categories such as porphyrins, coumarins, polycyclic aromatic hydrocarbons, benzothiazoles, oligonucleotide-based systems, and advanced drug delivery platforms (Table 3). Early-stage scaffold structures like porphyrins and anthraquinones laid the foundation for G4 recognition, while newer strategies—including dual-target inhibitors and nanotheranostic systems—are addressing longstanding challenges in specificity and drug delivery. Despite remarkable progress, challenges remain, including the trade-off between affinity and selectivity, ambiguity of mechanisms, and limited clinical validation. Future research should prioritize structure-guided ligand design, targeting specific environments, and integrated therapeutic strategies, particularly those that combine G4 stabilization with epigenetic regulation or immune activation. Ultimately, the successful clinical application of G4-targeting drugs will depend on resolving these issues and developing therapies with precise targeting capabilities and multifunctionality, thus fully exploiting the biological potential of G4 structures.

**Table 3.** Targeted DNA G-quadruplex compounds

Drugs	Chemical structure	Targets	Cancer type	Ref.
Tetra-Pt	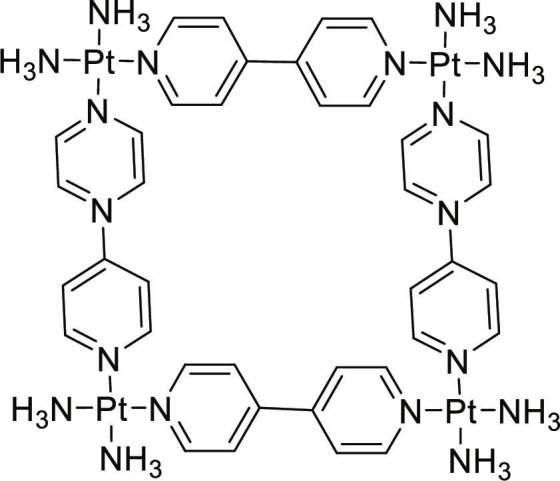	Telomeric G4	Pan-cancer	[120]
Pt-phen	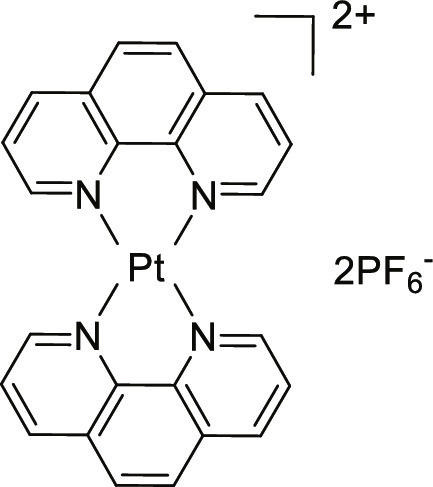	MYC G4-DNA	Breast cancer	[121]
L^1^-transpt	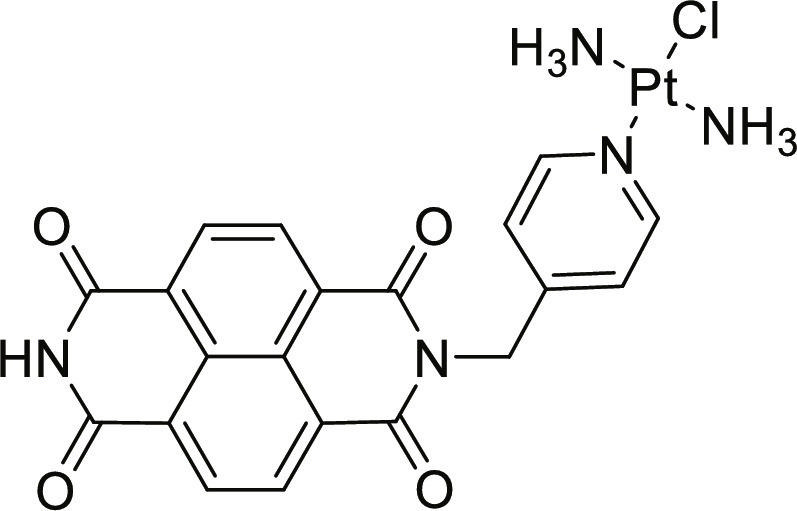	G4-DNA	Triple-negative breast cancer	[122]
Chiral RuII-PtII	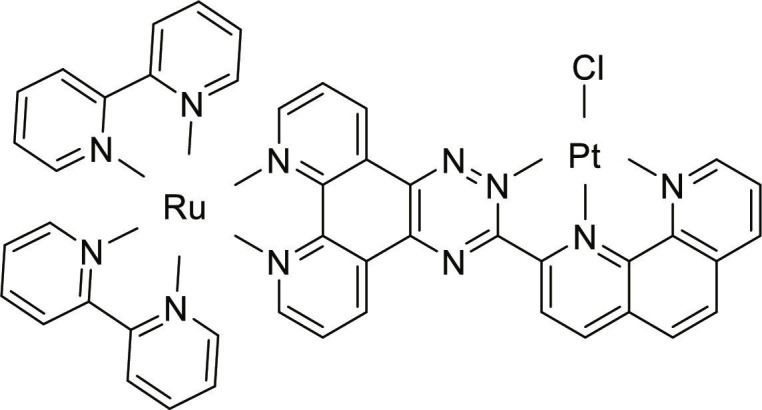	Telomeric G4	Lung cancer	[123]
RG260	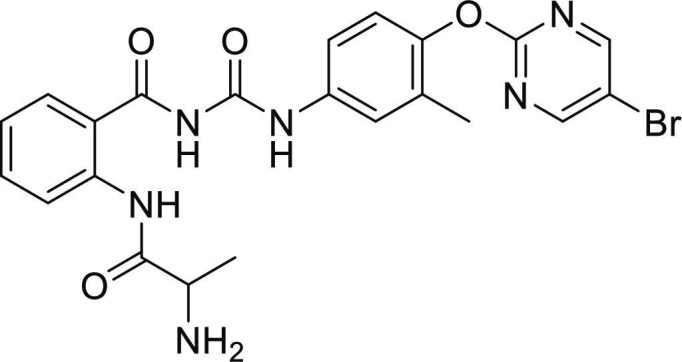	hTERT G4-DNA	Prostate cancer	[36]
BMPQ-1	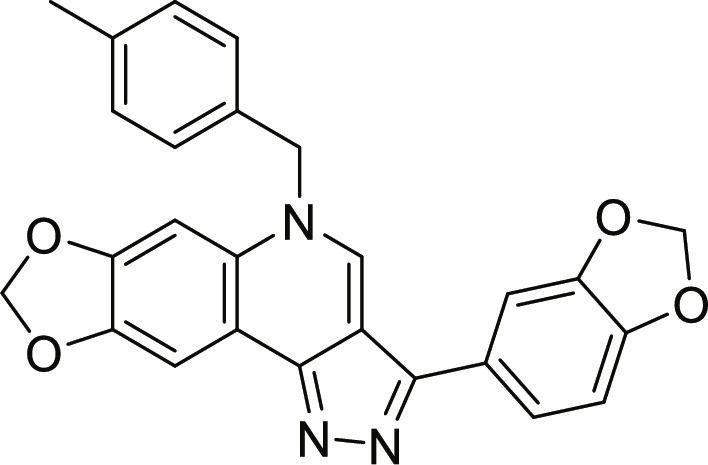	Telomeric multimeric G4-DNA	Lung cancer	[124]
QN-1	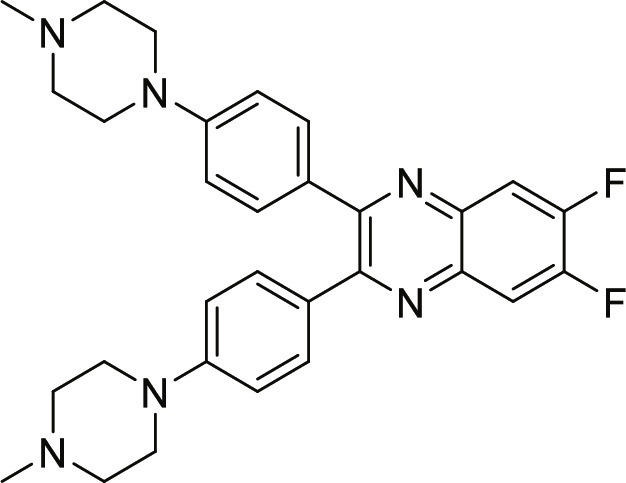	c-MYC G4-DNA	Triple-negative breast cancer	[125]
QN-1 Derivative	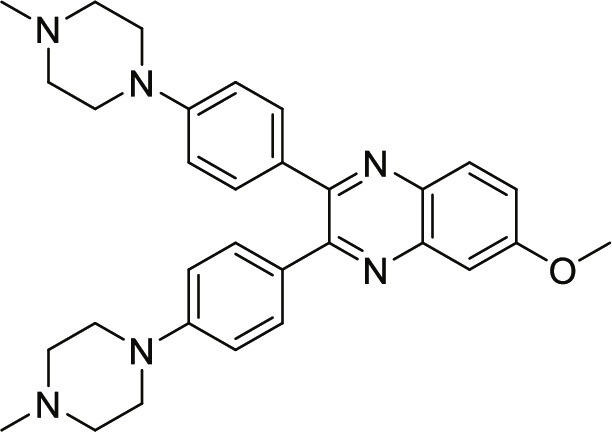	MYC G4-DNA	Triple-negative breast cancer	[126]
BMVC-8C3O	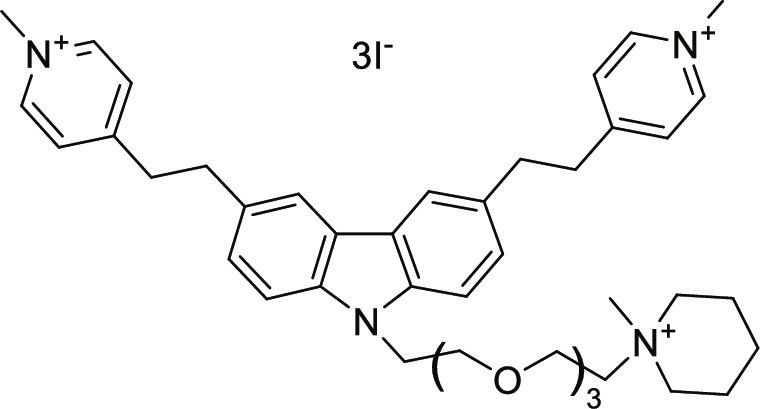	c-FOS G4-DNA	Non-small cell lung cancer	[127]
Q-Azo4F-C	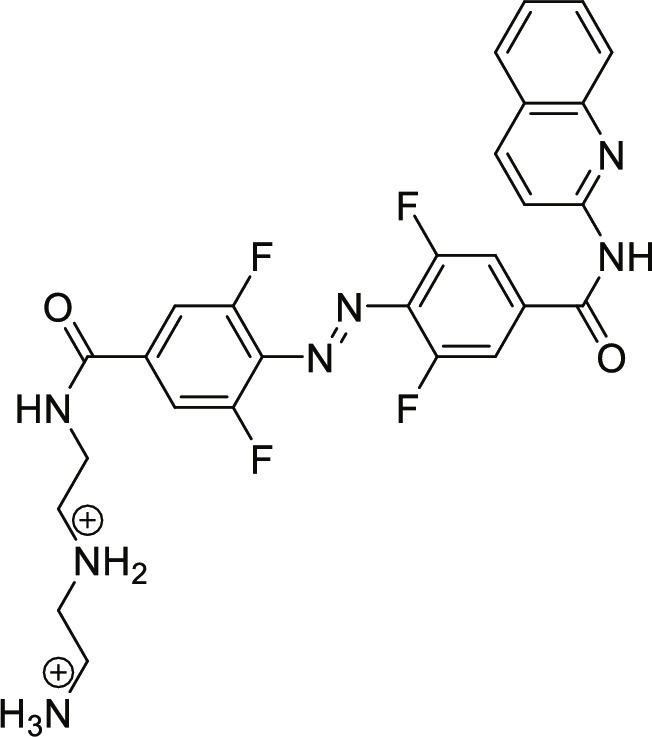	G4-DNA	Lung cancer	[128]
13d	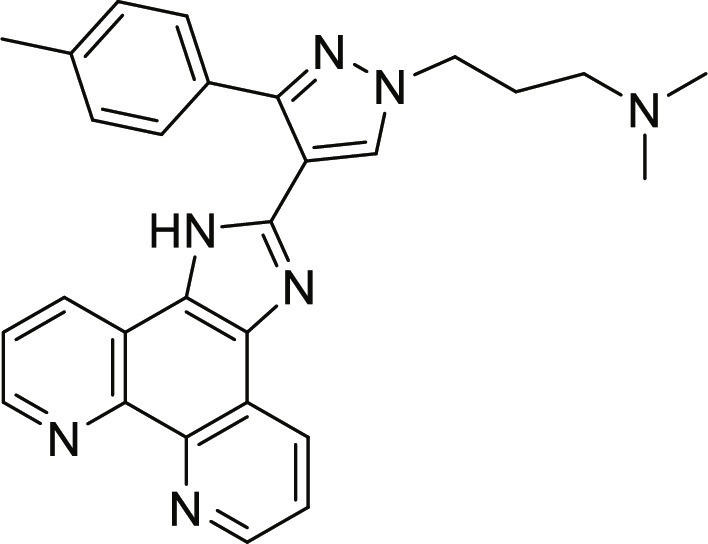	Telomeric G4	Gastric cancer	[129]
MY-8	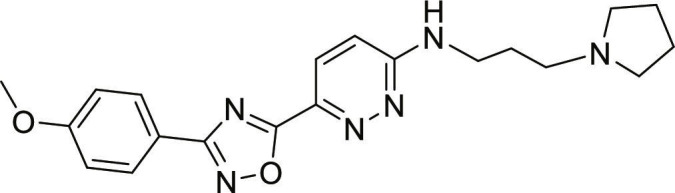	MYCN G4-DNA	Neuroblastoma	[130]
Compound 3	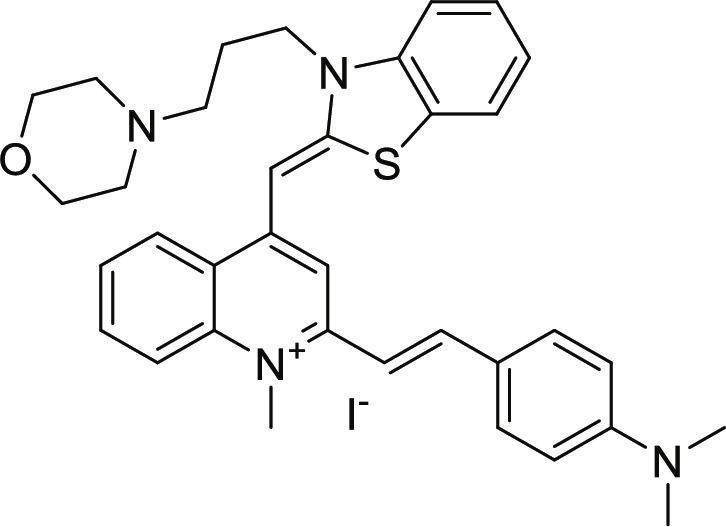	Telomeric G4	Pan-cancer	[154]
β-Thiomaltosyl-NDI-NMe2 12	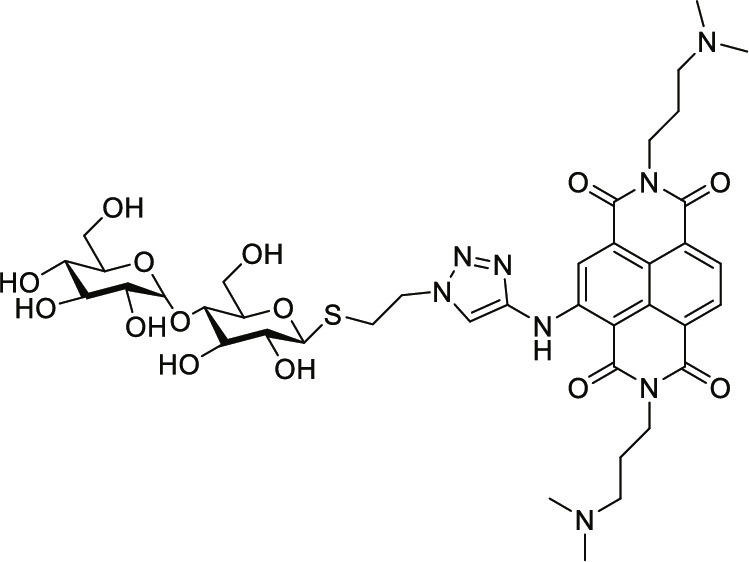	KRAS/MYC G4-DNA	Colorectal cancer	[132]
Naphthalimide-benzotriazole conjugates 3c	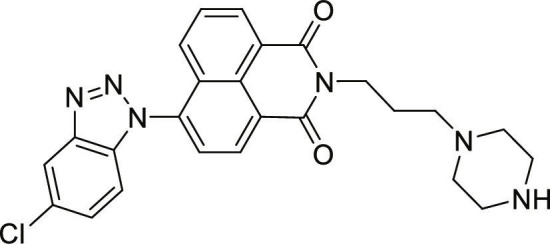	Bcl2-G4	Lung cancer	[133]
Benzothiophenonaphthalimides 7c	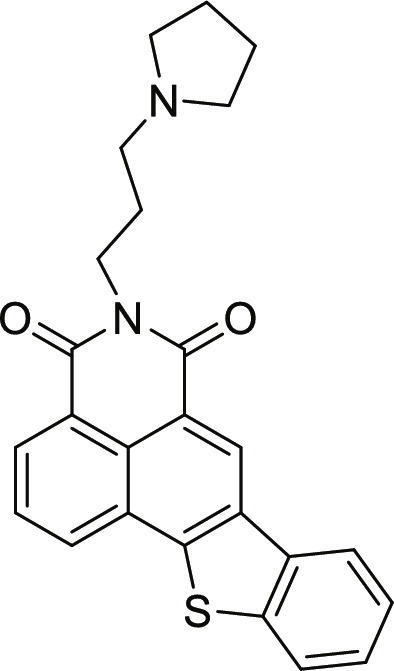	mtDNA G4	Human renal cell carcinoma	[134]
TGP18	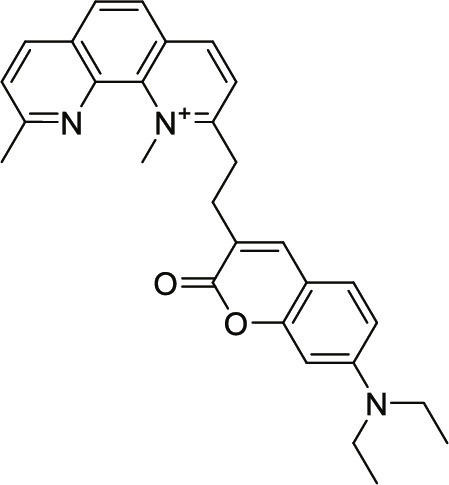	BCL-2 G4-DNA	Lung cancer	[137]
COP	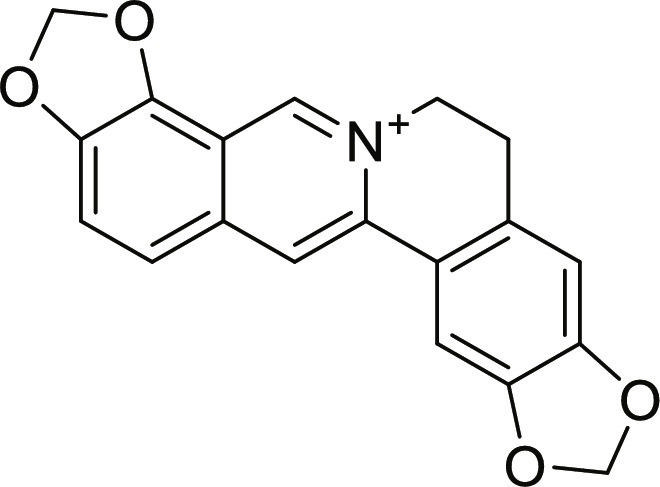	ATF4-G4	Pan-cancer	[138]
Compound 15	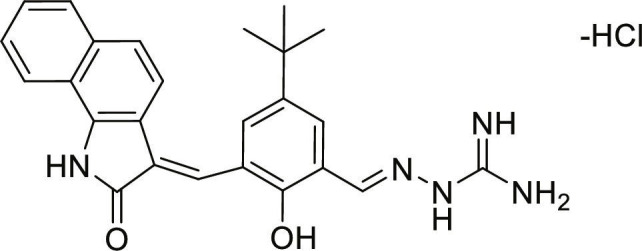	*Tel23* G4-DNA	Osteosarcoma Cervical carcinoma	[141]
DIZ-3	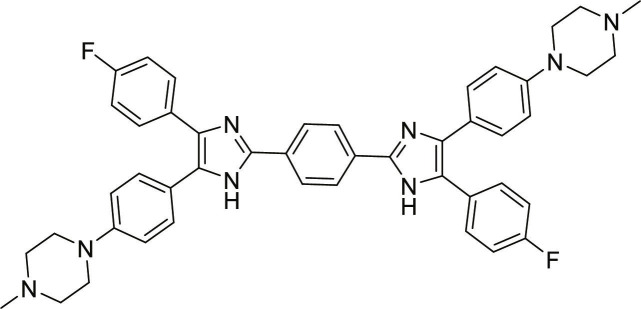	Telomeric multimeric G4-DNA	Osteosarcoma	[142]

### Targeting rG4s inhibits tumor progression

rG4 targeting represents a transformative approach in anticancer drug development, offering enhanced selectivity and broader target coverage compared to conventional therapies. Dickerhoff et al. [149] identified PEQ, a novel quinoline-based ligand that exhibits specific binding to MycG4. Structural analyses revealed that parallel G4 recognition involves flexible flanking residues adopting sequence-specific conserved conformations, providing a structural framework for rational rG4-targeted drug design. The therapeutic potential of rG4 targeting is further augmented through photodynamic combination strategies. Ferino et al. [150] developed cationic porphyrins (CPs) that selectively bind KRAS/NRAS mRNA rG4 structures, where photoirradiation triggers ROS generation to destabilize rG4 conformations, disrupt oncogenic metabolic pathways, and induce apoptosis in RAS-driven malignancies. Complementing this approach, Chen et al. [151] engineered the near-infrared photosensitizer BAMA, which incorporates an acridine-derived donor–π–acceptor system, enabling hypoxia-resistant superoxide anion production, specific rG4 recognition, and immunogenic cell death induction in immunologically cold tumors. These systems demonstrate how photodynamic rG4 modulation combines spatial precision with multimodal anticancer mechanisms, achieving both structural interference (rG4 destabilization) and biochemical effects while minimizing off-target toxicity through localized photoactivation (Table 4).

**Table 4.** Targeted RNA G-quadruplex compounds

Drugs	Chemical structure	Targets	Cancer type	Ref.
PEQ	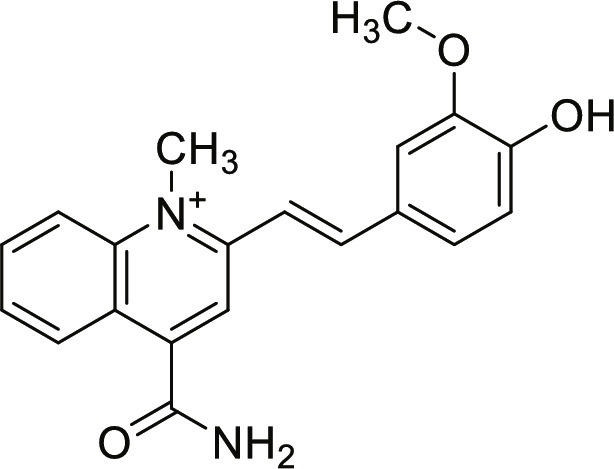	c-MYC G4-RNA	/	[149]
CPs	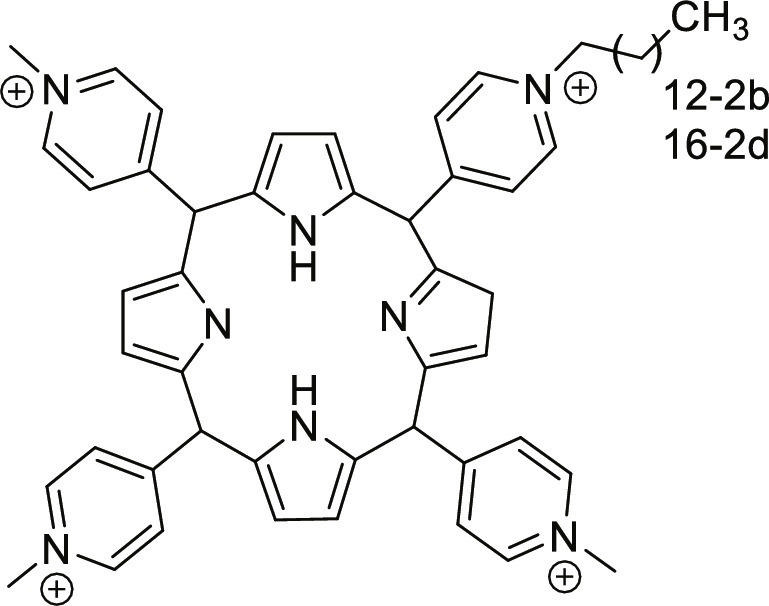	KRAS, NRAS G4-RNA	Pancreatic ductal adenocarcinoma	[150]
BAMA	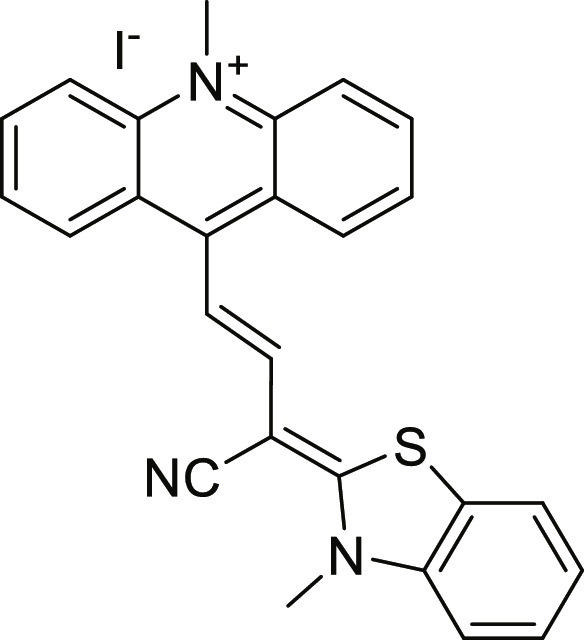	G4-RNA	Breast cancer	[151]

In addition to conventional G4 stabilizers, substantial advancements have been made in developing sophisticated G4 ligand–drug delivery systems. A notable breakthrough is the inducible ribonuclease targeting chimera (RIBOTAC) strategy developed by Zhang et al. [152], which conjugates an rG4 binder with a caged ribonuclease recruiter. This system exhibits tumor microenvironment-responsive activation, where the removal of the caging group triggers the recruitment of RNase L to specifically degrade G4-containing RNAs, thereby inducing programmed apoptosis for precise tumor elimination. The simultaneous incorporation of near-infrared (NIR) fluorescent G4 ligands enables real-time therapeutic monitoring. These platforms illustrate how rG4-targeted therapies achieve spatial precision through structure-specific molecular recognition, localized photoactivation, tumor microenvironment-responsive activation, and integrated diagnostic capabilities. The field is now transitioning from basic ligand development to sophisticated theranostic systems that address clinical challenges like tumor heterogeneity and drug resistance. However, challenges remain, including the dynamic nature of RNA structures, unresolved controversies surrounding stabilization versus destabilization strategies, and limited in vivo validation. Emerging technologies such as RIBOTAC and NIR-responsive systems offer promising solutions; however, further optimization is required for clinical translation. Future efforts must prioritize structure-based ligand design, transcriptome-wide target validation, and integration with immunotherapy and precision delivery systems.

### G4-targeted agents: Clinical progress and therapeutic horizons

G4-targeted therapeutic agents have transitioned from molecular probes to clinically relevant drug candidates, reflecting a broader paradigm shift in anticancer drug development toward noncanonical nucleic acid structures. Unlike conventional cytotoxic or protein-targeted therapies, G4 ligands operate at the structural level of the genome and transcriptome, thereby modulating oncogenic transcription, replication stress, and RNA processing.

**Fig. 6.**
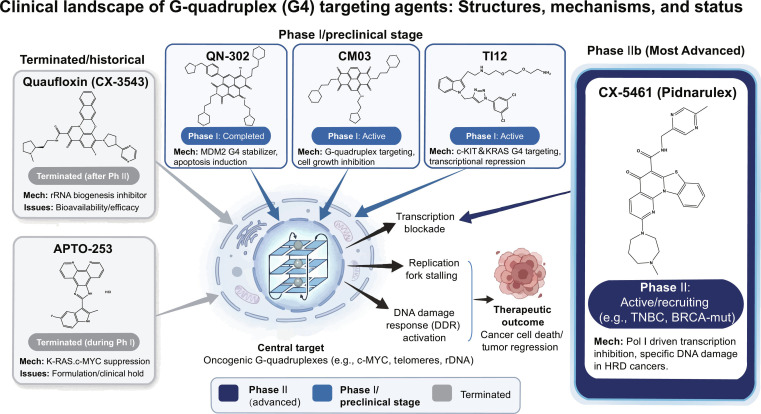
Clinical landscape of G4-targeting agents: Structures, mechanisms, and status G4-targeting agents are currently at different stages of clinical development and are categorized according to their status (terminated, preclinical, phase I, or advanced phase II). Agents such as quarfloxin (CX-3543) and APT-253 are highlighted for their historical development and associated challenges. Active agents, including QN-302 and CM03, exhibit promising mechanisms involving G4 stabilization and inhibition of cell growth, whereas CX-5461 (Pidnarulex) is undergoing advanced clinical evaluation targeting RNA polymerase I transcription. Key therapeutic outcomes associated with these agents include transcriptional blockade, replication fork stalling, and activation of DNA damage response pathways, ultimately leading to tumor regression.

The results presented in the accompanying table highlight various G4-targeted agents that have demonstrated promising safety profiles and tolerability, many of which are currently in preclinical or clinical stages (Table 5). The modes of action for these agents are diverse, encompassing transcriptional blockade and inhibition of cell growth, indicating their potential effectiveness against a variety of cancer types. For instance, Pidnarulex (CX-5461), a candidate in ongoing phase II clinical trials, targets RNA polymerase I, exhibiting antitumor activity in specific populations. Additionally, other agents such as QN-302 and CM03 are actively involved in clinical trials, targeting G4 structures in pancreatic and breast cancers. These agents show median inhibitory concentration (IC₅₀) values indicative of their potential to improve therapeutic efficacy, providing new options for the treatment of these malignancies (Fig. 6).

**Table 5.** G4-targeted drugs

Target/pathway	Drugs	Structures	Cancer types	Mode of action	Research model	Clinical protocol	Safety evaluation	Research and development stage	PMID
G4/TOP2B	Pidnarulex/CX-5461	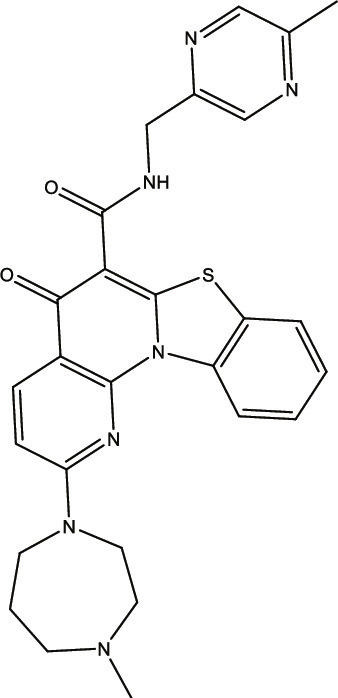	Advanced hematologic cancers	RNA polymerase I transcription inhibitor	G4 modulator TOP2B inhibitor	Once every 3 weeks MTD of 170 mg/m^2^	Well-tolerated	Phase I/II clinical trial NCT number:02719977	[139]
G4	QN-302/SOP-1812	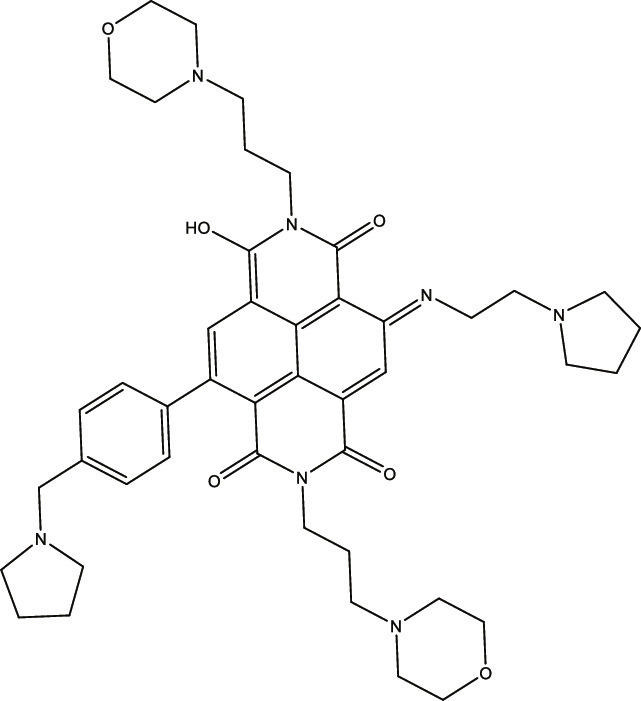	Pancreatic ductal adenocarcinoma	G-quadruplex inhibitor	G-quadruplex inhibitor	Intravenous infusion, once weekly for 3 weeks repeated every 4 weeks	Well-tolerated	Phase I clinical trial NCT number:06086522	[155]
G4	c-Myc G-quadruplex inhibitor X3	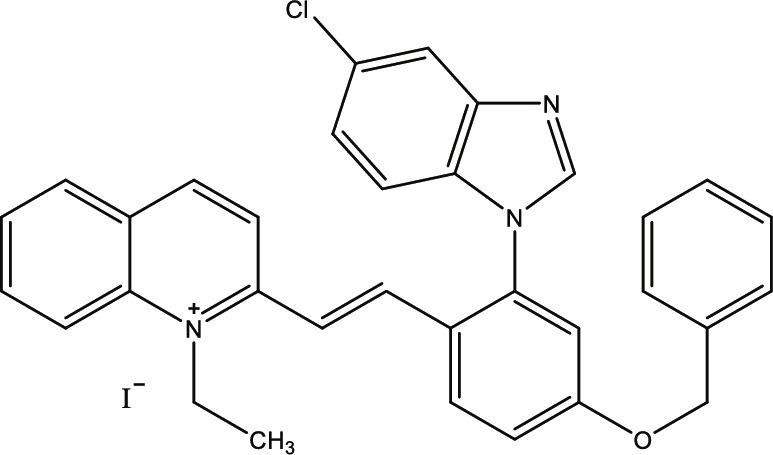	Breast cancer	c-Myc G-quadruplex inhibitor	G-quadruplex inhibitor	IC_50_ = 13.89 μM (MCF-7) IC_50_ = 9.855 μM (4T1)	Well-tolerated	Preclinical stage	[156]
G4	R1	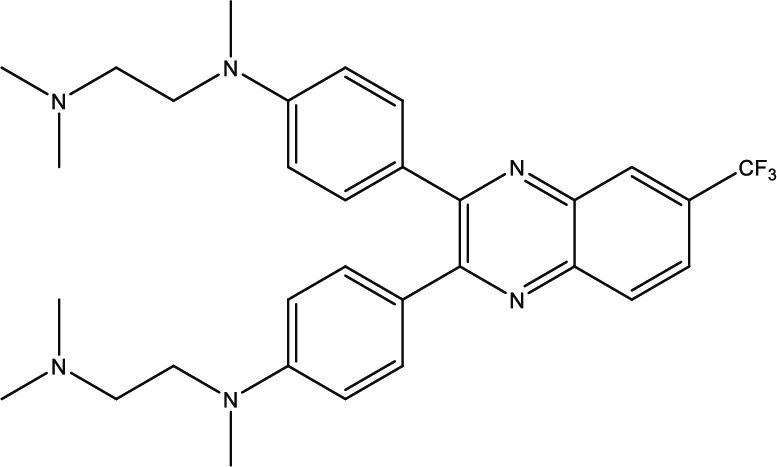	Neoplasms	G-quadruplex inhibitor	G-quadruplex inhibitor	10 mg/kg, once every other day, continuous administration for 2 weeks	Well-tolerated	Preclinical stage	[157]
G4	TI12	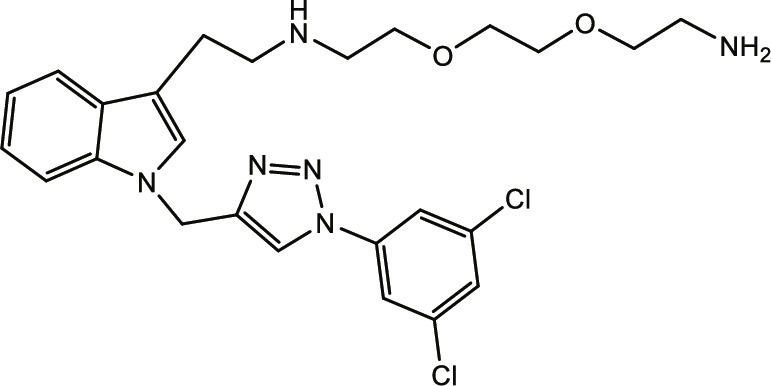	Leukemia	G-quadruplex inhibitor	G-quadruplex inhibitor	Two dose groups: 10 and 20 mg/kg; intratumoral injection, twice a week, continuous administration for 24 d	Well-tolerated	Preclinical stage	[158]
G4	c-Myc G-quadruplex inhibitor W11	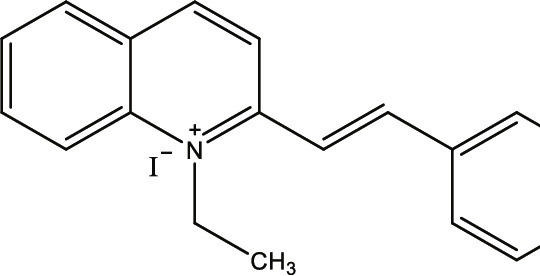	Breast cancer	c-Myc G-quadruplex inhibitor	G-quadruplex inhibitor	IC_50_ = 2.009 μM (MCF-7) IC_50_ = 1.961 μM (4T1)	Well-tolerated	Preclinical stage	[156]
G4	Compound 9	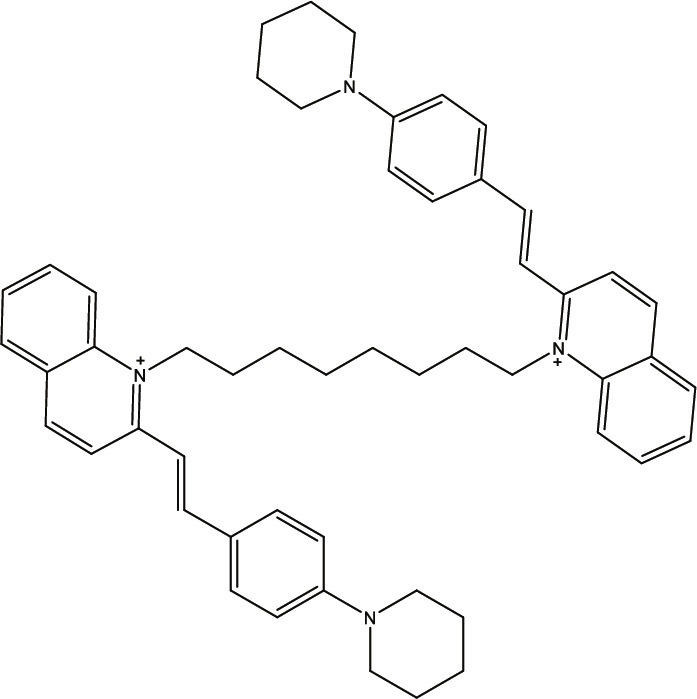	Colorectal cancer	G4-mtDNA modulator	G4 modulator	5 mg/kg, once every 2 d for a total of 16 d	Well-tolerated	Preclinical stage	[159]
G4	Pyridine bis-quinazoline derivatives 7c	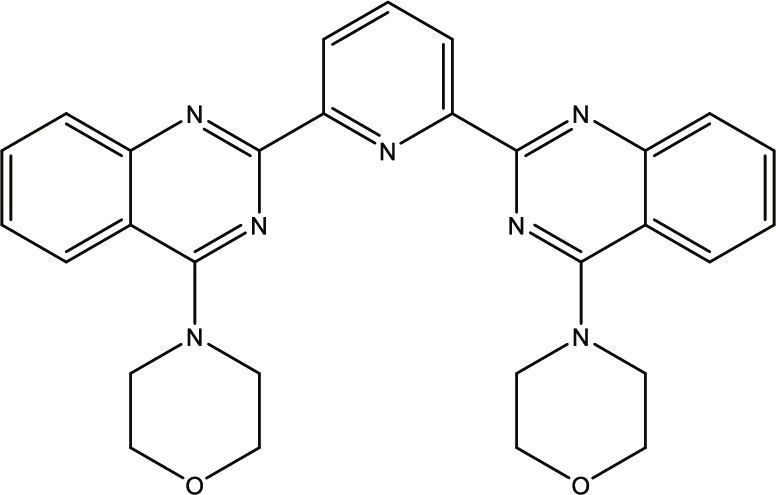	Neoplasms	G4-DNA stabilizer	G4 modulator	IC_50_ = 0.54 μM (HCT-8) IC_50_ = 0.3 μM (HepG2)	Well-tolerated	Preclinical stage	[160]
G4	CM03	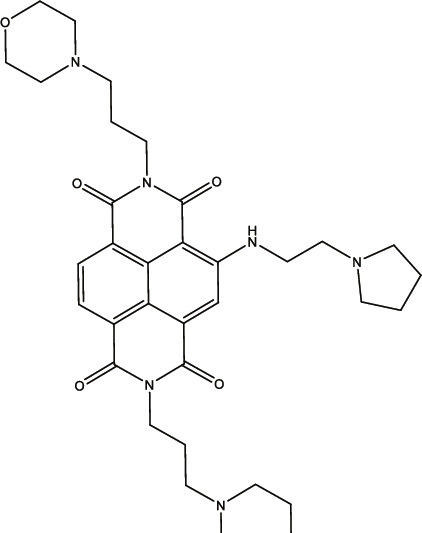	Pancreatic ductal adenocarcinoma	G4-DNA stabilizer	G-quadruplex inhibitor	15 mg/kg	Well-tolerated	Preclinical stage	[155]
G4	Pyridine bis-quinazoline derivatives 7n	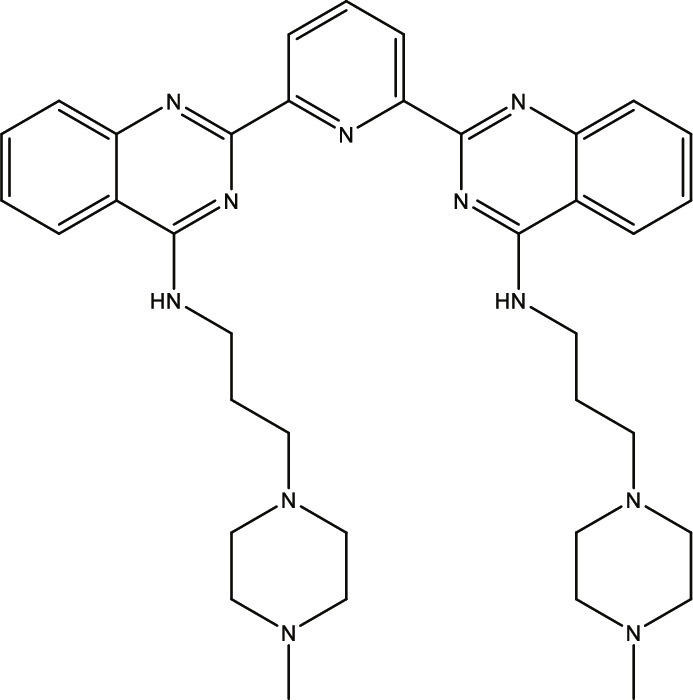	Neoplasms	G4-DNA stabilizer	G4 modulator	IC_50_ = 1.0 μM (HCT-8) IC_50_ = 0.3 μM (HepG2)	Well-tolerated	Preclinical stage	[160]
G4	Pyridine bis-quinazoline derivatives 7b	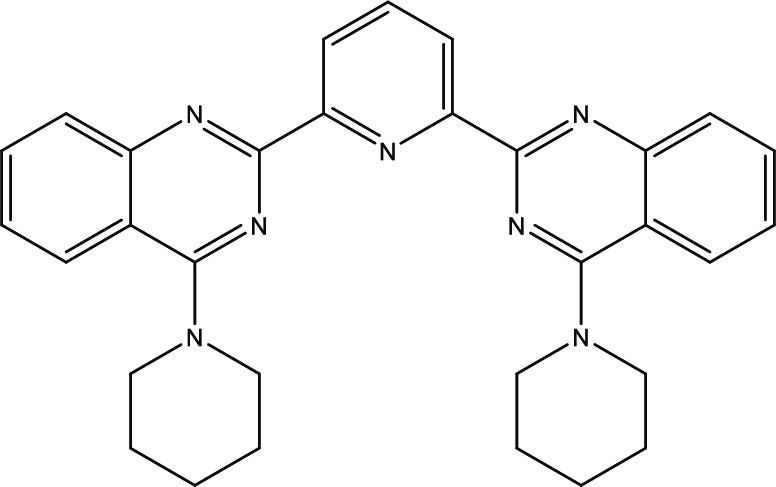	Neoplasms	G4-DNA stabilizer	G4 modulator	IC_50_=0.43 μM (HCT-8) IC_50_ = 0.3 μM (HepG2)	Well-tolerated	Preclinical stage	[160]
G4	Pyridine bis-quinazoline derivatives 7d	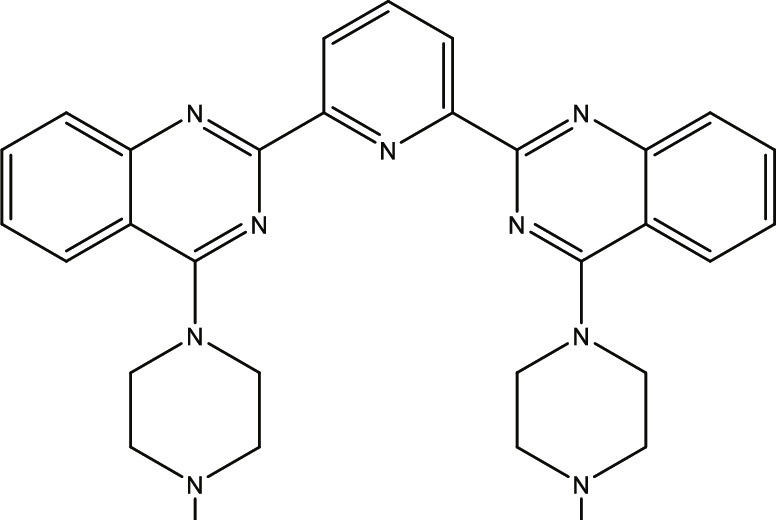	Neoplasms	G4-DNA stabilizer	G4 modulator	IC_50_ = 0.6 μM (HCT-8) IC_50_ = 0.42 μM (HepG2)	Well-tolerated	Preclinical stage	[160]
G4	SOP1247	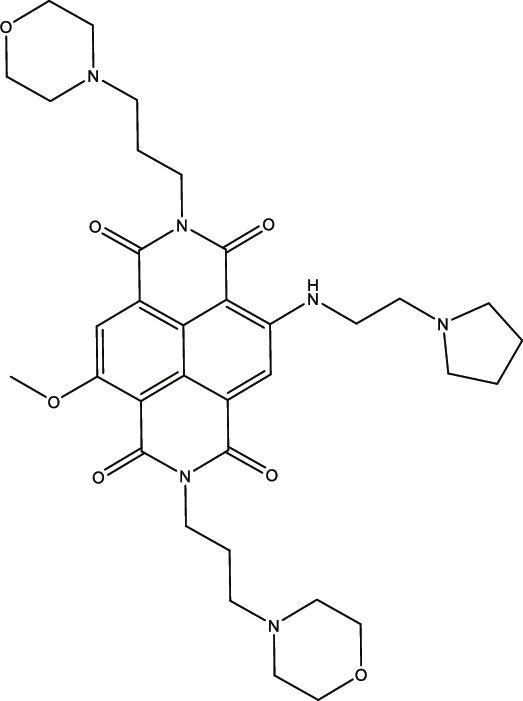	Pancreatic ductal adenocarcinoma	G4-DNA stabilizer	G-quadruplex inhibitor	GI_50_ = 13.8 nM (MIA-PaCa2) GI_50_ = 15.7 nM (PANC-1) GI_50_ = 38.8 nM (CAPAN-1) GI_50_ = 20.5 nM (Bx-PC3) (administration duration: 96 h)	Well-tolerated	Preclinical stage	[155]
G4 Top1	Compound 2	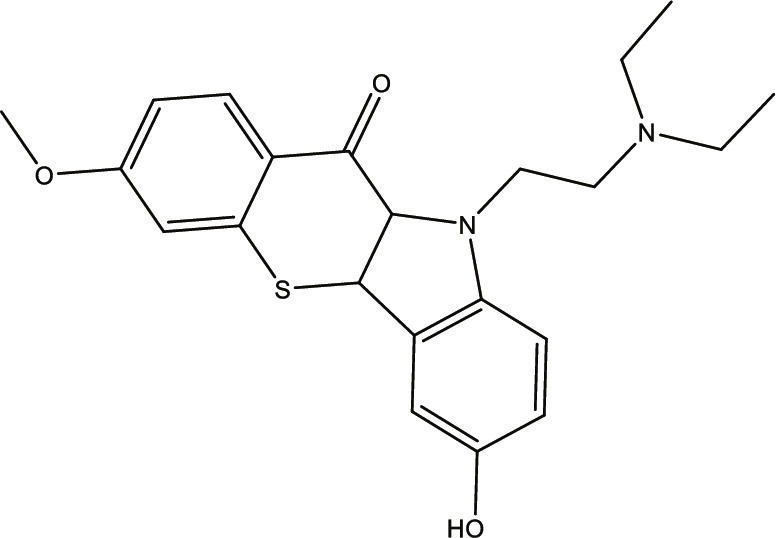	Colonic cancer	Inhibit G4\Top1	G-quadruplex inhibitor Top1inhibitor	2.5 μM inhibit Topo I GI_50_ = 0.389 μM (MOLT-4) GI_50_ = 0.675 μM (SW620) GI_50_ = 13.4 μM (SK-OV-3)	Well-tolerated	Drug discovery	[161]
G4 x Telomerase	CN116655703	\	Myelodysplastic syndromes, neoplasms, hemic and lymphatic diseases	\	\	\	Well-tolerated	Drug discovery	\
G4	G4 modulators/iron chelating agents modulators, compound 16	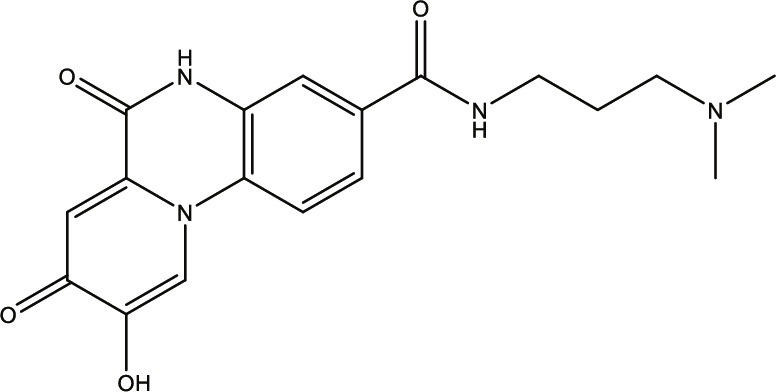	Neoplasms	Chelation of the labile iron pool; G4-DNA stabilizer	G4 modulators, iron chelators	IC_50_ = 30.4 μM	Well-tolerated	Drug discovery	[162]

Overall, G4-targeted drugs exhibit substantial anticancer potential and favorable preclinical performance, warranting considerable attention. Despite preclinical support, the clinical landscape remains selective rather than broad, with a limited number of candidates entering clinical trials. Future progress will depend on addressing critical academic controversies, improving structural specificity targeting, and integrating G4 therapeutics into the precision oncology framework. Ultimately, the success of G4-targeted therapies will hinge on transitioning from generalized genomic targeting to microenvironment-aware, biomarker-driven strategies.

## Conclusion and Prospects

G4s, distinctive noncanonical secondary structures prevalent in both the genome and transcriptome, have emerged as critical regulators in cancer initiation, progression, and therapeutic intervention. Recent advancements in high-throughput sequencing, structural biology, and chemical biology have revealed the remarkable tumor specificity of G4s, demonstrating their multifaceted roles in modulating oncogene expression, regulating telomere maintenance programs, orchestrating DDR pathways, and facilitating immune evasion. These mechanistic insights establish G4s as novel biomarkers for early cancer detection, precision prognostic indicators, and promising targets for molecularly guided therapies.

In cancer early detection, G4s exhibit substantial potential as molecular sentinels, particularly in proto-oncogene promoters (e.g., *MYC*, *KRAS*, *BCL-2*, and *c-KIT*), where G4-forming sequences function as “molecular switches” for oncogenic activation. Cutting-edge research reveals that aberrant G4 stabilization or dynamic dysregulation in these regions occurs during early tumorigenesis, often preceding conventional genetic alterations such as point mutations or copy number variations. The development of high-sensitivity G4-specific detection platforms now enables noninvasive early screening through characterization of cell-free G4 signatures in liquid biopsies. For prognostic evaluation, G4 formation is strongly correlated with tumor aggressiveness. High-throughput G4 mapping technologies have established that global G4 levels in tumor tissues substantially correlate with clinical stage progression, metastatic potential, and treatment resistance development.

Research on G4s in cancer development and therapy presents new promise and opportunities for the field of oncology. The enrichment of G4 structures in oncogene promoters and mtDNA enhances the tumor specificity of targeted therapies. Furthermore, synergistic effects may be achieved by combining G4-targeting approaches with immunotherapy, epigenetic modulators, or radiotherapy. Future research should harness artificial intelligence for the computational design of therapies, employing machine learning algorithms to predict G4-forming sequences and design highly selective ligands. The development of multifunctional integrated systems—specifically, “theranostic” platforms that combine G4 targeting, light-controlled release, and immune activation—represents a promising direction. While considerable challenges remain, advances in multidisciplinary research are expected to empower G4-targeted therapeutic strategies to play a pivotal role in precision oncology in the near future, ultimately improving therapeutic outcomes and quality of life for cancer patients.

Despite substantial advances in G4-targeted cancer therapy, several critical challenges warrant consideration. Primarily, the in vivo structure–function relationship of G4 remains incompletely elucidated, particularly due to its transient characteristics and sensitivity to cellular contexts. Secondly, the persistent affinity-selectivity paradox endures: Ligands exhibiting high G4-binding affinity often demonstrate inadequate genomic or transcriptomic specificity, resulting in nontarget effects. Thirdly, mechanistic obscurity complicates clinical translation, as therapeutic outcomes frequently cannot be definitively attributed to direct G4 modulation versus secondary stress responses such as replication stress or DNA damage. Fourth, the absence of robust biomarkers and patient stratification approaches restricts the incorporation of G4-targeted therapies within the precision oncology framework. Lastly, challenges in drug delivery and bioavailability, particularly concerning RNA-targeting systems and large molecular platforms, remain substantial obstacles. Looking forward, artificial intelligence possesses transformative potential for accelerating the discovery of selective ligands, while advanced genome-wide mapping technologies continue to refine our understanding of context-dependent G4 landscapes. Additionally, the integration of G4-targeting strategies with emerging therapeutic modalities—particularly immunotherapy and PARP inhibition—holds promise, as rationally designed combinations may exploit synthetic lethality and overcome resistance mechanisms limiting the durability of monotherapies.

In summary, G4 structures have transitioned from fundamental biological curiosities to pivotal targets in precision oncology. They demonstrate transformative potential across 3 key clinical domains: the discovery of early-warning biomarkers, the refinement of prognostic stratification systems, and the development of novel targeted therapeutics. As our understanding of the dynamic G4 regulatory network deepens, and with the integration of enabling technologies such as artificial intelligence, G4-targeted strategies are poised to emerge as a third pillar of cancer therapy—complementing kinase inhibitors and immunotherapies. This advancement promises to catalyze a paradigm shift toward an integrated era of cancer management that encompasses prediction, intervention, and, ultimately, curative strategies.
